# Mathematical modeling and cost-effective intervention strategies for diabetes management: A data-driven and numerical analysis approach

**DOI:** 10.1371/journal.pone.0339463

**Published:** 2026-01-16

**Authors:** S. Nivetha, Mini Ghosh

**Affiliations:** Department of Mathematics, School of Advanced Sciences, Vellore Institute of Technology, Chennai, Tamil Nadu, India; Parahyangan Catholic University: Universitas Katolik Parahyangan, INDONESIA

## Abstract

Diabetes mellitus is a chronic, non-communicable disease that continues to pose a major global health burden. Effective management strategies require not only clinical interventions but also robust modeling frameworks to guide public health decisions. In this study, we develop a compartmental model to describe the dynamics of diabetes under both pharmacological and non-pharmacological interventions. The model is solved using a Nonstandard Finite Difference (NSFD) scheme, which preserves key properties such as positivity and boundedness, and demonstrates superior stability compared to classical numerical methods (RK4 and Forward Euler). The model parameters are estimated by fitting to annual diabetes prevalence data from the United States (2000–2022). A global sensitivity analysis using Partial Rank Correlation Coefficients (PRCC) identifies the most influential parameters driving disease dynamics. To evaluate intervention strategies, the model is extended with three time-dependent control measures, followed by a cost-effectiveness analysis to assess their relative efficiency. Our findings highlight the critical parameters influencing diabetes progression and suggest optimal intervention strategies that balance effectiveness with economic feasibility. These results provide valuable insights for improving diabetes management and support evidence-based public health planning.

## Introduction

Diabetes mellitus is a chronic metabolic disorder and a major global public health concern. Characterized by sustained elevated blood glucose levels, diabetes can lead to serious complications such as cardiovascular diseases, kidney failure, neuropathy, and vision impairment if not properly managed. According to the World Health Organization (WHO) and the International Diabetes Federation (IDF), approximately one in nine adults worldwide is currently living with diabetes, with the majority residing in low and middle income countries [1,2]. The global prevalence of diabetes has been rising steadily, driven by demographic transitions, urbanization, and sedentary lifestyles. This growing burden not only affects individual well-being but also places substantial pressure on healthcare systems. As emphasized by the IDF, improving the lives of people living with diabetes and preventing the disease in those at risk remains a key public health mission.

Numerous factors contribute to the development of diabetes, among which obesity is one of the most significant [3,4]. Excess body weight increases the risk of insulin resistance, impairing the body’s ability to regulate blood glucose effectively. This interaction often creates a feedback loop where obesity and diabetes coexist, worsening health outcomes. This close relationship commonly termed “diabesity” is driven by shared metabolic defects such as insulin resistance and hormonal imbalances [5]. In particular, central obesity markedly increases the risk of both diabetes and cardiovascular disease. Effective management typically requires a combination of lifestyle modifications and, in some cases, pharmacological interventions [6]. Genetic factors, along with environmental and lifestyle influences, also play a vital role in the development of diabetes. Genetic epidemiology explores how gene variants and environmental factors interact to shape diabetes risk, highlighting the complex nature of both type 1 and type 2 diabetes [7,8]. Additionally, epigenetic mechanisms further influence disease susceptibility throughout life.

To better understand the complex mechanisms underlying diabetes, epidemiology plays an important role. Beyond infectious disease modeling, mathematical models are increasingly used to study the mechanisms of non-communicable diseases such as diabetes, cancer, etc. To address the growing burden of diabetes, mathematical modeling has emerged as a valuable tool for understanding disease mechanisms and informing public health strategies. For instance, recent models have simulated pancreatic islet adaptation to insulin resistance and provided realistic long-term predictions of disease progression [9]. Reviews of the field underscore the growing importance of such models and the need for deeper mechanistic insights into diabetes and its complications [10]. Other models have explored transitions from diabetes to its complication stages, demonstrating the potential for cost-effective intervention strategies [11]. These efforts reinforce the value of mathematical modeling in guiding research, prevention, and treatment strategies for diabetes.

In the study of nonlinear dynamical systems, particularly when analytical solutions are not feasible, numerical methods serve as essential tools for exploring system behavior. Standard schemes such as the Forward Euler and fourth-order Runge–Kutta (RK4) methods are widely used due to their simplicity and effectiveness in approximating solutions of ordinary differential equations. These methods have been successfully applied in modeling various infectious diseases, including HIV-AIDS, COVID-19, and historical influenza, providing meaningful insights into disease dynamics [12–14]. While RK4 offers improved accuracy over Euler for small step sizes, both methods may encounter limitations especially when applied to discrete-time compartmental models failing to preserve crucial properties such as positivity, boundedness, and long-term stability [15,16].

To overcome these challenges, the Nonstandard Finite Difference (NSFD) method has been proposed as a robust alternative. NSFD schemes are specifically designed to maintain key qualitative features of the continuous system, including positivity, conservation laws, and dynamic consistency, regardless of step size [17,18]. Moreover, NSFD methods have been shown to accurately capture complex nonlinear behaviors, such as backward and Hopf bifurcations, making them particularly suitable for biological and epidemiological modeling [19]. Applications of NSFD to diseases like hepatitis B, dengue, diarrhea, and malaria have further demonstrated its superior performance over traditional methods [15,19–21].

In recent years, optimal control theory has been applied to diabetes models to identify time-dependent intervention strategies that minimize disease burden while considering associated costs [22]. Complementing this, cost-effectiveness analysis enables evaluation of these strategies from an economic perspective, assisting policymakers in the efficient allocation of limited healthcare resources [23]. Furthermore, models have increasingly incorporated stochastic effects, environmental factors such as endocrine-disrupting chemicals, and behavioral influences to better capture disease dynamics and complexities. These studies apply Pontryagin’s Maximum Principle to identify effective control strategies that mitigate disease progression and related complications. Research highlights the importance of addressing co-morbid behaviors such as alcohol consumption and obesity while accounting for treatment variability and environmental exposures [24–27]. Numerical simulations validate these approaches, providing actionable insights for disease management and policy planning. In addition, optimal control and cost-effective frameworks have also been extensively used beyond diseases for example in suppressing nonlinear computer virus transmission [28]. Similar approaches have also been successfully implemented in TB behavioral control [29], HCV elimination strategy evaluation in prisons [30], malaria–VL co-infection control [31], and even dual addiction dissemination modeling [32].

Additionally, recent work has focused on combining awareness initiatives with treatment and psychological follow-up to effectively control type 2 diabetes. By applying optimal control methods with time-dependent controls for awareness and treatment, these models identify cost-efficient strategies for reducing disease prevalence. Computational simulations confirm that integrating education and therapy offers both significant health benefits and economic feasibility [33,34]. Finally, fractional-order diabetes models incorporating insulin therapy and awareness controls have been developed to optimize treatment strategies. Stability and cost-effectiveness analyses of these models, supported by numerical simulations, demonstrate the benefits of combined interventions [35].

In contrast to existing diabetes models, the present study integrates both pharmacological and non-pharmacological (lifestyle-based) interventions together with obesity-induced diabetes progression and the transition between treatment types within a unified dynamical framework. Existing mathematical models on diabetes are relatively limited in number, and most of them consider general treatment effects without distinguishing between these intervention strategies or validating the theoretical findings with real-time data. To address this gap, our model is calibrated using observed prevalence data, providing a quantitative validation of the system dynamics and enabling an evidence-based assessment of intervention outcomes.

From the mathematical and computational perspective, a tailored Nonstandard Finite Difference (NSFD) scheme is developed to ensure positivity, boundedness, and dynamic consistency of numerical solutions features not explicitly guaranteed in most earlier diabetes models. Furthermore, we integrate Partial Rank Correlation Coefficient (PRCC) based global sensitivity analysis with optimal control and cost effectiveness assessments to quantitatively evaluate and compare intervention strategies. To the best of our knowledge, this comprehensive integration of mechanistic modeling, real-time data calibration, numerically stable discretization, sensitivity analysis, optimal control, and cost-effectiveness evaluation has not been reported previously in the context of diabetes dynamics.

The paper is structured as follows. Sect 1 presents the formulation of the mathematical model together with the existence of equilibrium points and the stability analysis. Sect 2 describes the data fitting using the maximum likelihood method, followed by numerical simulations of the diabetes model, including the PRCC-based sensitivity analysis. Sect 3 introduces the NSFD scheme together with its convergence, consistency, and error analysis. Sect 4 focuses on the optimal control and cost-effectiveness analyses to evaluate the efficiency of different intervention strategies. Sect 5 provides the results and discussion. Finally, Sect 6 concludes the study with the main findings.

## 1 Mathematical model formulation

Diabetes is one of the main chronic diseases that affect global health. It typically arises in individuals due to factors such as lifestyle changes, genetic predisposition, and other metabolic issues. In its early stages, diabetes can often be managed or even reversed through non-pharmacological interventions such as a healthy lifestyle, regular exercise, yoga, and proper diet. In more advanced stages, pharmacological treatment, including insulin therapy, becomes necessary. Individuals who manage their diabetes effectively by following a healthy routine often inspire others to adopt similar habits, a phenomenon supported by peer influence studies [36,37]. In our model, this behavioral effect is captured by the transition from the diabetic compartment (*D*) to the non-pharmacological treatment compartment (*T*_*N*_), represented using a mass-action term, $\frac{\psi T_{N}D}{N}$. This term reflects motivation through peer support and social influence, driving individuals to adopt lifestyle-based management strategies even though diabetes is not an infectious disease and cannot be transmitted from person to person.

By incorporating these considerations, we formulated a mathematical model using a compartmental framework. In this model, the total human population is divided into five compartments: susceptible individuals (*S*), individuals with diabetes (*D*), individuals undergoing pharmacological treatment (*T*_*P*_), which includes insulin administration and medication, individuals undergoing non-pharmacological treatment (*T*_*N*_), involving healthy lifestyle interventions such as yoga, walking, and dietary control [38,39], and restrained individuals (*R*). The restrained compartment represents individuals whose diabetes is effectively controlled or in remission as a result of sustained treatment and behavioural changes. While not biologically cured, these individuals maintain glucose levels within a regulated range and are at reduced risk of diabetes-related complications. The interactions among these compartments capture the dynamics of disease progression, treatment response, and transitions into long-term controlled states within the population.

1. Λ is the recruitment rate of individuals entering the susceptible compartment.
2. *β* is the baseline rate at which individuals develop diabetes.
3. $\alpha_{1}$ is the proportion of individuals who develop diabetes due to overweight.
4. The term $\alpha_{2}$ represents the proportion of individuals who develop diabetes due to genetic or other complications.
5. *η* is a modification parameter such that η>1, indicating that overweight individuals have a higher likelihood of developing diabetes compared to those with genetic or other factors. The total rate of diabetes development is given by $\beta (\eta \alpha_{1}+\alpha_{2})$, where $\alpha_{1}+\alpha_{2}=1$.
6. *ω* is the relapse rate the rate at which individuals who were under control (restrained) return to the diabetic state.
7. *ψ* is the rate at which individuals initiate non-pharmacological treatment, including interventions such as lifestyle modification, diet, and physical activity.
8. *δ* is the rate at which individuals move to pharmacological treatment, such as oral medications or insulin therapy.
9. *γ* is the rate at which individuals under treatment (pharmacological ) achieve controlled or managed diabetes. *θ* denotes the rate at which individuals in the non-pharmacological treatment compartment transition to the restrained (controlled) diabetes state.
10. $\omega_{1}$ and $\omega_{2}$ represent the rates at which individuals discontinue non-pharmacological and pharmacological treatments, respectively, and return to the *D* compartment.
11. *ϕ* denotes the proportion of individuals under non-pharmacological treatment who transition to pharmacological treatment due to health deterioration.
12. *μ* is the natural death rate.
13. $\sigma_{1}$, $\sigma_{2}$, and $\sigma_{3}$ represent the excess mortality rates due to diabetes-related complications in individuals, and in those in the pharmacological and non-pharmacological treatment compartments, respectively.

**Fig 1:**
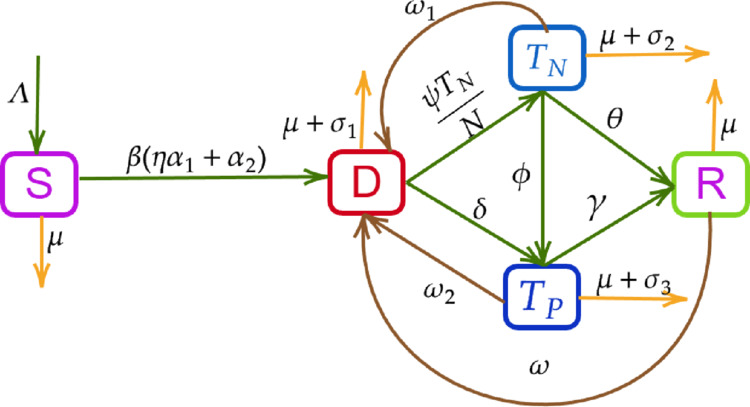
A schematic diagram illustrating the diabetes model with five compartments: S denotes susceptible individuals, D represents individuals with diabetes, $T_{N}$ corresponds to those receiving non-pharmacological treatment, $T_{P}$ indicates individuals under pharmacological treatment and R stands for those who have controlled or restrain.

Based on the above assumptions and the schematic representation in Fig 1, we develop the following mathematical model for diabetes.


$$\frac{dS}{dt}=\Lambda -\beta (\eta \alpha_{1}+\alpha_{2})S-\mu S,$$

$$\frac{dD}{dt}=\beta (\eta \alpha_{1}+\alpha_{2})S+\omega R+\omega_{1}T_{N}+\omega_{2}T_{P}-\frac{\psi T_{N}D}{N}-(\delta +\mu +\sigma_{1})D,$$

$$\frac{dT_{N}}{dt}=\frac{\psi T_{N}D}{N}-(\theta +\mu +\sigma_{2}+\omega_{1}+\phi )T_{N},$$

$$\frac{dT_{P}}{dt}=\delta D+\phi T_{N}-(\gamma +\omega_{2}+\mu +\sigma_{3})T_{P},$$

$$\frac{dR}{dt}=\gamma T_{P}+\theta T_{N}-(\mu +\omega )R.$$ (1)

### 1.1 Positivity and the bounded nature of the solution

From the system, we obtain the following


$$\frac{dS}{dt}{|}_{S=0}=\Lambda >0,\;\;\frac{dD}{dt}{|}_{D=0}=\beta (\eta \alpha_{1}+\alpha_{2})S+\omega R+\omega_{1}T_{N}+\omega_{2}T_{P}\geq 0,\;\;\frac{dT_{N}}{dt}{|}_{T_{N}=0}=0,$$

$$\frac{dT_{P}}{dt}{|}_{T_{P}=0}=\delta D+\phi T_{N}\geq 0,\;\;\frac{dR}{dt}{|}_{R=0}=\gamma T_{P}+\theta T_{N}\geq 0$$

All the rates mentioned above are non-negative at the bounding planes. Therefore, starting from the interior of the non-negative bounded cone $R_{+}^{5}$, we will stay within this cone. This is because the vector direction is inward on all the bounding planes. Consequently, the solutions of the model will remain non-negative. Additionally, according to the compartmental model, the total human population $N=S+D+T_{N}+T_{P}+R$


$$\frac{dN}{dt}=\Lambda -\mu N-(\sigma_{1}D+\sigma_{2}T_{N}+\sigma_{3}T_{P}),$$

This gives,


$$\underset{t\to \infty }{lim sup}N\leq \frac{\Lambda }{\mu }.$$

Therefore, every solution $S(t),D(t),T_{N}(t),T_{P}(t),R(t)$ is bounded by $\frac{\Lambda }{\mu }$. This gives us the biologically feasible region of the compartmental model (1) by the below positively invariant set:


$$\Gamma =\left\{(S,D,T_{N},T_{P},R)\in R_{+}^{5}:0\leq S,D,T_{N},T_{P},R\leq \frac{\Lambda }{\mu }\right\}.$$

### 1.2 Existence of equilibrium points

In the proposed diabetes model, three biologically feasible equilibrium points are identified. These equilibria are determined by setting the right-hand sides of the system of differential equations to zero and solving for the steady-state values of the variables. Each equilibrium corresponds to a distinct diabetes scenario and provides insight into the long-term dynamics of diabetes within the population.

1. **Diabetes-Free Equilibrium (DFE)**: This equilibrium represents a scenario in which no individual in the population is affected by diabetes. It is given by
  $$E_{0}=(S^{*},0,0,0,0),\;\text{where}\;S^{*}=\frac{\Lambda }{\beta (\eta \alpha_{1}+\alpha_{2})+\mu }.$$
2. **Diabetic Equilibrium without Non-Pharmacological Treatment**: This equilibrium, denoted by
  $$E_{1}=(S^{*},D^{*},0,T_{P}^{*},R^{*}),$$
  characterizes the state where individuals have developed diabetes and are undergoing pharmacological treatment, but no non-pharmacological interventions are present. The components of the unique equilibrium are given by:
  $$S^{*}=\frac{\Lambda }{\beta (\eta \alpha_{1}+\alpha_{2})+\mu },\;D^{*}=\frac{A_{4}A_{5}A_{1}S^{*}}{\delta \gamma \mu +\delta A_{5}(\mu +\sigma_{3})+A_{4}A_{5}(\mu +\sigma_{1})},$$
  $$T_{N}^{*}=0,\;T_{P}^{*}=\frac{\delta D^{*}}{A_{4}},\;R^{*}=\frac{\gamma \delta D^{*}}{A_{4}A_{5}}.$$
3. **Endemic Equilibrium**: This equilibrium describes a situation in which all compartments are active, indicating persistent presence of the diabetes and its treatments in the population. It is given by
  $$E_{2}=(S^{*},D^{*},T_{N}^{*},T_{P}^{*},R^{*}),$$
  where:
  $$S^{*}=\frac{\Lambda }{\beta (\eta \alpha_{1}+\alpha_{2})+\mu },\;D^{*}=\frac{A_{3}N^{*}}{\psi },\;T_{N}^{*}=(\frac{A_{1}S^{*}+A_{8}D^{*}}{\psi D^{*}-A_{9}N^{*}})N^{*},$$
  $$T_{P}^{*}=\frac{\delta D^{*}+\phi T_{N}^{*}}{A_{4}},\;R^{*}=A_{6}D^{*}+A_{7}T_{N}^{*},$$
  $$N^{*}=\frac{A_{4}\psi S^{*}}{A_{4}\psi A_{9}+A_{4}A_{3}(1+\delta +A_{6})}+\frac{\left[(A_{4}+A_{4}A_{8}+\phi )(A_{1}\psi S^{*}+A_{8}A_{3})\right]}{A_{4}\psi A_{9}+A_{4}A_{3}(1+\delta +A_{6})(A_{3}-A_{9})}.$$
  *N*  is positive if $A_{3}>A_{9}$ and the constants *A*_*i*_ are defined as follows:
  $$A_{1}=\beta (\eta \alpha_{1}+\alpha_{2}),\;A_{2}=(\mu +\delta +\sigma_{1}),\;A_{3}=(\theta +\mu +\sigma_{2}+\omega_{1}+\phi ),$$
  $$A_{4}=(\gamma +\mu +\sigma_{3}+\omega_{2}),\;A_{5}=(\mu +\omega ),\;A_{6}=\frac{\gamma \delta }{A_{5}A_{4}},\;A_{7}=\frac{\phi +A_{4}\theta }{A_{5}A_{4}}.$$
  $$A_{8}=(\omega A_{6}+A_{2}+\frac{\omega_{1}\delta }{A_{4}}),\;A_{9}=(\omega_{1}+A_{7}\omega +\frac{\phi \omega_{2}}{A_{4}}).$$

### 1.3 Stability analysis

**Theorem 1.1.** *The Diabetes-Free Equilibrium is locally asymptotically stable under certain parameter restrictions.*

*Proof:* Proof of the theorem is available in S1 File. □

**Theorem 1.2.** *The equilibrium point *E**_*1*_ *(Diabetic Equilibrium without Non-Pharmacological Treatment) is locally asymptotically stable under certain conditions.*

*Proof:* Proof of the theorem is available in S1 File. □

**Fig 2:**
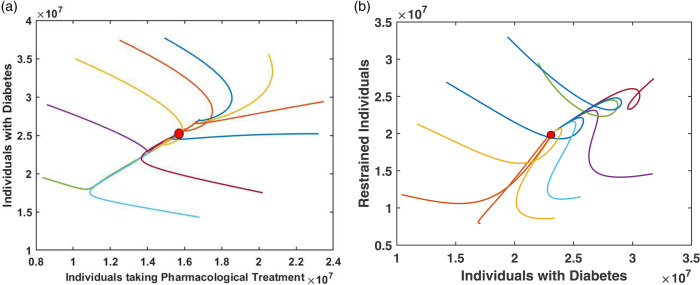
2D phase portraits showing the stability of the equilibrium point $E_{1}$ for the pairs (a) $\left(D,T_{P}\right)$ and (b) (D, R). Each trajectory is initiated from a different initial condition, and all trajectories converge to the same equilibrium point. This indicates local asymptotic stability of *E*_1_ in these two-dimensional projections.

**Fig 3:**
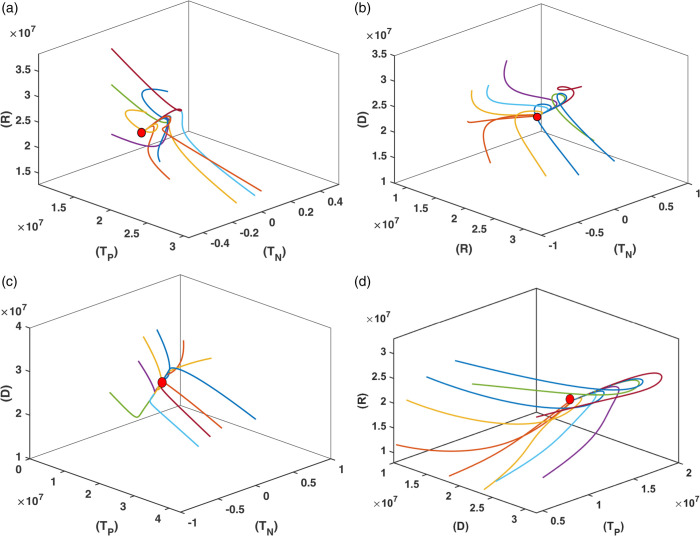
3D phase portraits illustrating the convergence of trajectories toward the equilibrium point $E_{1}$ for the combinations (a) (R, $T_{P},T_{N}$), (b) $\left(R,T_{N},D\right)$, (c) $\left(T_{P},T_{N},D\right)$ and (d) $\left(D,T_{P},R\right)$. Each plot displays ten trajectories starting from different initial conditions, with *T*_*N*_ = 0 initially, consistent with $E_{1}=(S^{*},D^{*},0,T_{P}^{*},R^{*})$. The consistent convergence in all subplots confirms the asymptotic stability of *E*_1_.

In Figs 2 and 3, we graphically examine the stability of the equilibrium point *E*_1_ (Diabetic Equilibrium without Non-Pharmacological Treatment) using 2D and 3D phase portraits, respectively. Fig 2 presents the 2D phase portraits for the pairs (*D*,*T*_*P*_) and (*D*,*R*). In both plots, ten trajectories are initiated from different initial conditions, and all trajectories converge to the same equilibrium point. Fig 3 displays 3D phase portraits for the variable combinations $\left(D,T_{P},T_{N}\right)$, (*R*,*T*_*P*_,*D*), $\left(R,T_{P},T_{N}\right)$, and (*R*,*T*_*N*_,*D*). Here as well, each plot starts from ten different initial conditions. Since the equilibrium point $E_{1}=(S^{*},D^{*},0,T_{P}^{*},R^{*})$ corresponds to the absence of non-pharmacological treatment, all trajectories begin with *T*_*N*_ = 0. Despite this, in all four 3D projections, the trajectories converge to the same equilibrium point. These results clearly demonstrate that the equilibrium point *E*_1_ (Diabetic Equilibrium without Non-Pharmacological Treatment) is asymptotically stable. All simulations for Figs 2 and 3 are performed using the parameter values listed in Table 1.

**Table 1:** Model parameters, their descriptions, values, and sources.

Parameter	Description	Value	Source
Λ	Constant recruitment rate	11/1000	[41]
*β*	Baseline diabetes incidence rate	0.1478	Estimated
*η*	Modification factor accounting for increased risk	1.0573	Estimated
$\alpha_{1}$	Rate of diabetes onset due to overweight	0.1346	Estimated
$\alpha_{2}$	Rate of diabetes onset due to genetic or other factors	0.8653	$1-\alpha_{1}$
*ω*	Relapse rate	0.31	[42]
*ψ*	Rate at which individuals initiate non-pharmacological treatment	0.7634	Estimated
*δ*	Rate at which individuals initiate pharmacological treatment	0.6832	Estimated
*θ*	Restrained rate from *T*_*P*_	0.0098	Estimated
$\omega_{1}$	Discontinuation rate of non-pharmacological treatment back to *D*	0.0235	Estimated
$\omega_{2}$	Discontinuation rate of pharmacological treatment back to *D*	0.5	[43]
*ϕ*	Fraction moving from non-pharmacological to pharmacological treatment	0.0235	Assumed
*γ*	Restrained rate from *T*_*N*_	0.4495	[44]
$\sigma_{1}$	Excess mortality rate due to diabetes-related complications from *D*(*t*)	0.013	[45]
$\sigma_{2}$	Excess mortality rate due to diabetes-related complications from *T*_*N*_(*t*)	0.2160	Assumed
$\sigma_{3}$	Excess mortality rate due to diabetes-related complications from *T*_*P*_(*t*)	0.1381	Assumed
*μ*	Natural death rate	0.0128	Demography

**Theorem 1.3.** *The equilibrium point *E**_*2*_ *(Endemic Equilibrium) is locally asymptotically stable under appropriate parameter conditions.*

*Proof:* Proof of the theorem is available in S1 File. □

**Fig 4:**
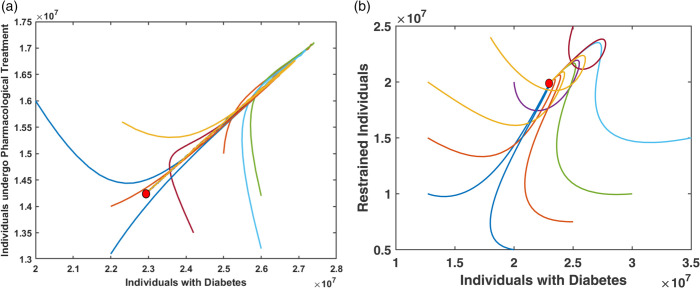
2D phase portraits showing the stability of the endemic equilibrium point $E_{2}$ for the variable pairs (a) $\left(D,T_{P}\right)$, and (b) (D, R). Each trajectory starts from a different initial condition and converges to the same equilibrium point, indicating asymptotic stability in these phase planes.

**Fig 5:**
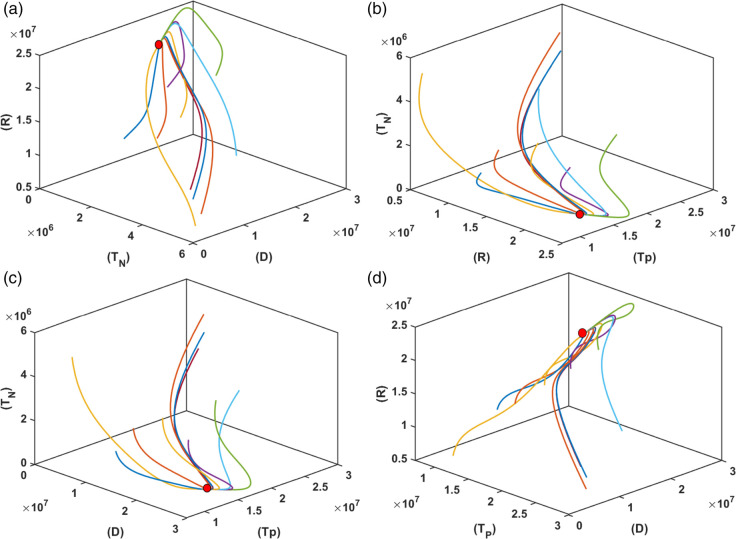
3D phase portraits illustrating the convergence of trajectories toward the endemic equilibrium point $E_{2}$ for various variable combinations: (a) $\left(R,T_{P},D\right)$, (b) $\left(R,T_{P},T_{N}\right)$, (c) $\left(D,T_{P},T_{N}\right)$, and (d) $\left(R,T_{N},D\right)$. Ten trajectories are shown in each plot, each starting from a different initial condition. The consistent convergence across all plots supports the asymptotic stability of *E*_2_.

Figs 4 and 5 illustrate the graphical analysis of the stability of the endemic equilibrium point *E*_2_ using both 2D and 3D phase portraits. In Fig 4, 2D phase portraits are shown for the variable pairs (*D*,*T*_*P*_), and (*D*,*R*). For each pair, trajectories originating from ten different initial conditions are plotted. In all cases, the trajectories converge to the same equilibrium point, indicating local stability in the corresponding phase planes. Fig 5 provides 3D phase portraits for various combinations of the model variables: $\left(D,T_{P},T_{N}\right)$, (*R*,*T*_*P*_,*D*), $\left(R,T_{P},T_{N}\right)$, and (*R*,*T*_*N*_,*D*). Each plot also includes ten trajectories starting from distinct initial conditions. Across all projections, the trajectories consistently converge to the endemic equilibrium point *E*_2_. These phase space analyses confirm that the endemic equilibrium *E*_2_ is asymptotically stable under the given model dynamics. All simulations for Figs 4 and 5 are performed using the parameter values listed in Table 1

## 2 Numerical simulation

To validate the analytical findings, numerical simulations are conducted by fitting the model to real-world diabetes data and estimating the relevant parameters. Sensitivity analysis using PRCC is performed, and the effects of parameter variations are examined through time evolution and contour plots.

### 2.1 Data fitting

Fitting the diabetes model to real-world data plays a crucial role in understanding diabetes dynamics and informing effective control strategies. In this study, the Maximum Likelihood Estimation (MLE) method is employed to fit the model and estimate its parameters, with a detailed description of the method provided in [40]. The dataset is obtained from the CDC Diabetes Surveillance System (https://gis.cdc.gov/grasp/diabetes/diabetesatlas-surveillance.html) and consists of annual U.S. diabetes data from 2000 to 2022. Using these 23 data points, we estimate seven key parameters of the model.

**Fig 6:**
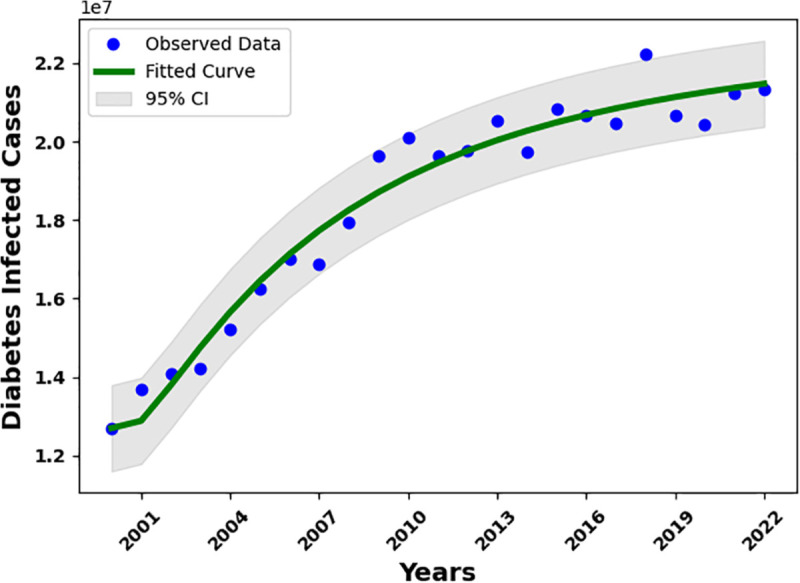
The diabetes model is fitted to the number of new cases, with a 95% confidence interval. Yearly diabetes data from the United States, covering the years 2000 to 2022, is used.

The estimated parameters in our model include *β*, *η*, $\alpha_{1}$, *ψ*, *δ*, *θ*, and $\omega_{1}$, which are obtained through the data-fitting procedure described earlier. The parameters $\omega_{2}$, *ω*, *γ*, and $\sigma_{1}$ are sourced from published literature, whereas *ϕ*, $\sigma_{2}$, and $\sigma_{3}$ are assumed based on biological reasoning and treatment progression patterns. The constant recruitment rate Λ and the natural death rate *μ* are treated as demographic constants. Here, “demography” refers specifically to the quantitative natural mortality rate *μ*, calculated from an average life expectancy of 78 years (i.e., μ=1/78≈0.0128 per year). The fitted curve is shown in Fig 6, and the complete set of parameter values is summarized in Table 1.

### 2.2 Sensitivity analysis using PRCC

The Partial Rank Correlation Coefficient (PRCC) sensitivity analysis assesses the influence of individual input parameters on the model’s output while controlling for the effects of other variables. This technique is especially valuable for complex, nonlinear models, as it helps identify the parameters that most strongly affect model behavior. A higher PRCC value indicates a greater sensitivity of the output to the corresponding parameter.

In this study, we performed 1000 simulations using U.S. datasets to determine which parameters significantly influence the variables: *D* (Individuals with Diabetes), *T*_*N*_ (Non-Pharmacological Treatment), *T*_*P*_ (Pharmacological Treatment), and *R* (Restrain). Parameters with absolute PRCC values exceeding 0.5 are considered to have a strong impact on these variables.

**Fig 7:**
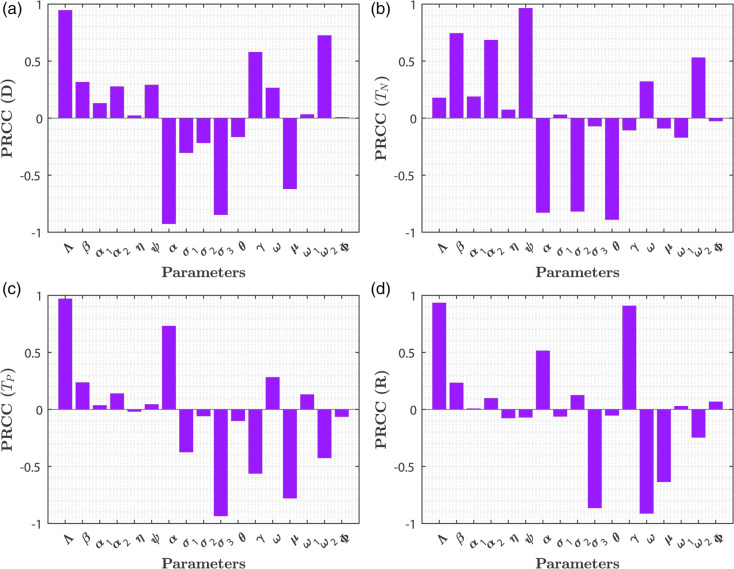
Partial Rank Correlation Coefficients (PRCC) for various model parameters with respect to: (a) individuals with diabetes, (b) individuals undergoing non-pharmacological treatment, (c) individuals undergoing pharmacological treatment, and (d) restrained individuals.

Fig 7(a) reveals that the parameters $\beta ,\;\alpha ,\;\sigma_{3},\;\gamma ,$ and $\omega_{2}$, are most influential for *D*. Similarly, Fig 7(b) shows that $\beta ,\alpha_{2},\psi ,\alpha ,\sigma_{2},\theta ,\omega$ significantly affect *T*_*N*_. In Fig 7(c), the parameters $\Lambda ,\alpha ,\sigma_{3},\gamma ,\mu$ are found to be important for (*T*_*P*_). Lastly, Fig 7(d) indicates that $\Lambda ,\alpha ,\sigma_{3},\gamma ,\omega ,$ and *μ* play a significant role in determining (*R*) for the US dataset.

### 2.3 Variation in model parameters and its effect on diabetes prevalence

This section presents the approximate numerical solutions of the model equations using fourth and fifth-order Runge-Kutta methods, implemented via MATLAB’s ode45 solver, with parameter values provided in Table 1. The numerical simulations provide a clear understanding of the system’s temporal dynamics. In addition, they serve to confirm the theoretical findings and illustrate how changes in critical parameters influence the behavior of the model.

**Fig 8:**
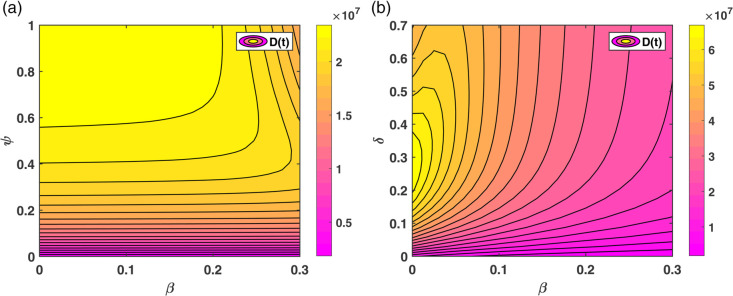
Contour plots showing the effect of parameter combinations on the diabetic population D(t): (a) β×ψ∈(0,0.1)×(0,1) and (b) β×δ∈(0,0.1)×(0,0.1).

The contour plot in Fig 8(a) illustrates that the number of individuals in the diabetes compartment D(t) increases with a rise in *β* (the baseline rate at which individuals develop diabetes), and decreases as *ψ* (the rate at which individuals initiate non-pharmacological treatment) increases. Fig 8(b) similarly shows that D(t) increases with a rise in *β* while it decreases as *δ* (the rate at which individuals initiate pharmacological treatment) increases. This implies that effective implementation of both non-pharmacological interventions (e.g., lifestyle modifications) and pharmacological treatments can substantially reduce the diabetes burden in the population, even when the incidence rate remains high.

**Fig 9:**
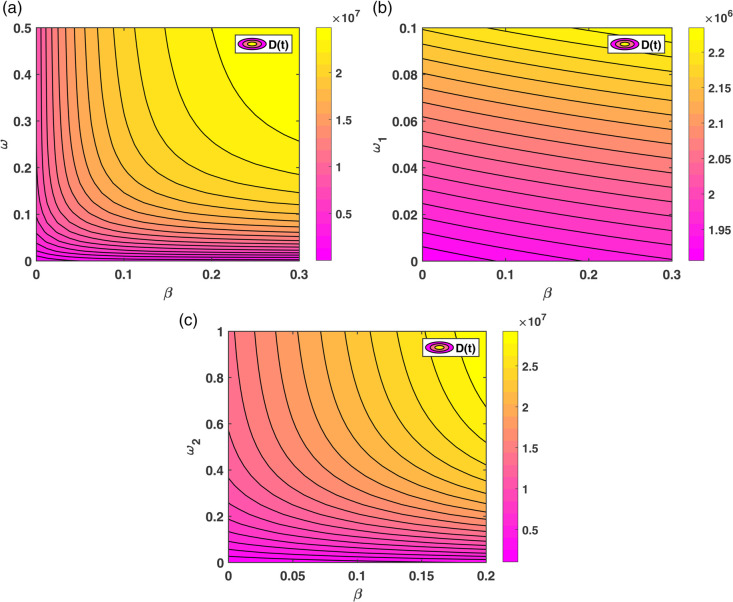
Contour plots showing the combined effect of the transmission rate β and relapse parameters on the diabetic population D(t). (a) (β×ω)∈[0,0.3]×[0,0.5] , where *ω* is the relapse rate from Restrain back to Diabetes, (b) $\left(\beta \times \omega_{1})\in [0,0.3]\times [0,0.1\right]$, where $\omega_{1}$ is the failure rate of non-pharmacological treatment and (c) $\left(\beta \times \omega_{2})\in [0,0.2]\times [0,1\right]$, where $\omega_{2}$ is the failure rate of pharmacological treatment.

The contour plots in Fig 9 clearly indicate that the relapse parameters *ω* (relapse from restrain back to diabetes), $\omega_{1}$ (failure of non-pharmacological treatment), and $\omega_{2}$ (failure of pharmacological treatment) have a substantial impact on increasing the diabetes population. Therefore, effective control and reduction of these relapse/failure rates can significantly reduce the burden of diabetes in the population.

**Fig 10:**
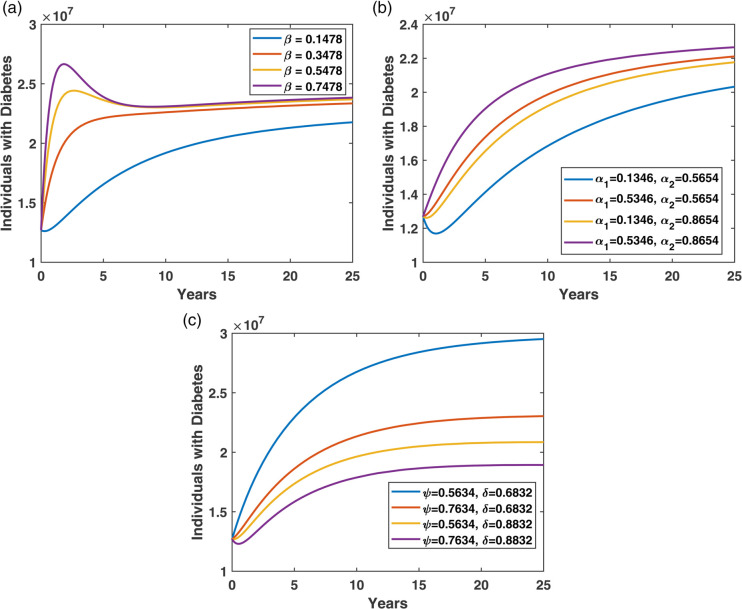
Time variation plots showing the effect of key parameters on individuals with diabetes: (a) the baseline rate at which individuals develop diabetes β, (b) risk factors $\alpha_{1}$ and $\alpha_{2}$ and (c) treatment rates ψ and δ.

The time variation plots in Fig 10 illustrate the dynamics of the individuals in diabetes compartments over time. Fig 10(a) shows that as the parameter *β* increases, the number of individuals developing diabetes also rises. This indicates a strong positive correlation between *β* and the incidence of diabetes. Higher values of *β* may reflect factors such as increased lifestyle-related risks or a lack of preventive measures.

Fig 10(b) illustrates the impact of the parameters $\alpha_{1}$ (the proportion of individuals who develop diabetes due to overweight) and $\alpha_{2}$ (the proportion who develop diabetes due to genetic or other complications). The figure indicates that, compared to $\alpha_{2}$, $\alpha_{1}$ plays a more significant role in increasing the number of diabetes cases, highlighting the greater influence of overweight and lifestyle-related factors.

Fig 10(c) shows the effect of treatment parameters *ψ* (rate at which individuals initiate non-pharmacological treatment) and *δ* (rate at which individuals move to pharmacological treatment). The results suggest that *δ* is more effective than *ψ* in reducing the impact of diabetes, emphasizing the stronger role of pharmacological interventions in managing the condition.

**Fig 11:**
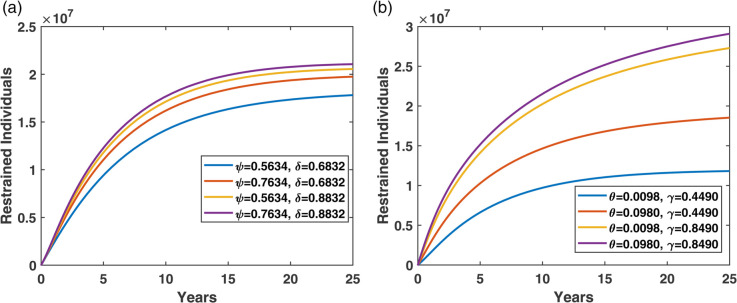
Effect of treatment initiation rates (ψ, δ) and restrain rates (θ, γ) on restrained compartment.

Fig 11(a) shows the effect of treatment parameters *ψ* (the rate at which individuals initiate non-pharmacological treatment) and *δ* (the rate at which individuals move to pharmacological treatment). The results suggest that *δ* is more effective than *ψ* in promoting restraining the progression of diabetes, emphasizing the stronger role of pharmacological interventions in managing the condition.

Fig 11(b) demonstrates how variations in the restrain rates *θ* (diabetes controlled from the *T*_*N*_ compartment) and *γ* (diabetes controlled from the *T*_*P*_ compartment) influence the temporal evolution of the restrained population. As either parameter increases, the peak of restrained individuals rises and occurs earlier, reflecting accelerated diabetes control dynamics. The scenario with the highest values of both parameters (θ=0.0980, γ=0.8490) yields the earliest and most pronounced peak in the restrain (controlled) compartment. These results highlight the importance of enhancing the transition processes in both *T*_*N*_ and *T*_*P*_ compartments to improve overall diabetes control outcomes.

**Fig 12:**
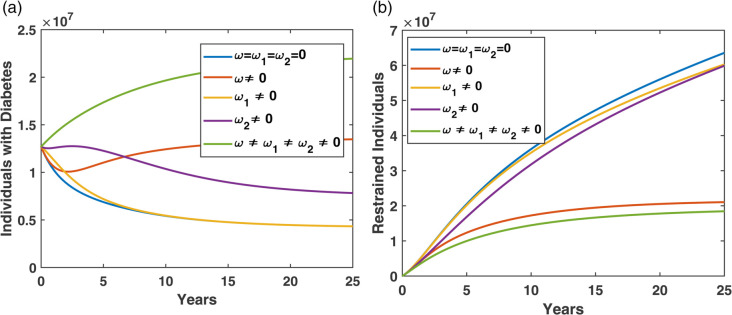
Effect of relapse (ω) and treatment failure ($\omega_{1},\omega_{2}$) on restrained individuals and diabetic prevalence.

In Fig 12, we analyse the influence of relapse and treatment failure rates on diabetes progression and diabetes controlled(restrain) outcomes. Here, *ω* denotes relapse from the restrained compartment back to the diabetes compartment, $\omega_{1}$ represents failure from the non-pharmacological treatment compartment, and $\omega_{2}$ represents failure from the pharmacological treatment compartment.

Fig 12(a) presents the dynamics of the diabetes compartment. When all relapse and failure pathways are absent $\left(\omega =\omega_{1}=\omega_{2}=0\right)$, the diabetic population remains comparatively lower. Introducing relapse alone (ω≠0) already produces a notable increase in the diabetic burden, and when both failure rates $\omega_{1}$ and $\omega_{2}$ are also included, the number of individuals with diabetes becomes significantly higher. Among the three mechanisms, relapse (ω) contributes the strongest increase in diabetic prevalence compared to the treatment failure mechanisms.

Fig 12(b) shows the dynamics of the restrained compartment. The restrained population is highest when relapse and treatment failure are absent. The inclusion of relapse alone reduces the restrained class, and the reduction becomes severe when both treatment failure pathways are active together. Thus, both relapse and treatment failure mechanisms weaken diabetes controlled outcomes, and relapse has a more pronounced negative impact compared to the other two failure rates.

For simulations, we used ω=0.31, $\omega_{1}=0.0235$, $\omega_{2}=0.5$, and all other parameter values are provided in Table 1.

## 3 Nonstandard finite difference (NSFD) method

### 3.1 Development of a nonstandard finite difference scheme

In this section, we construct and analyze a Nonstandard Finite Difference (NSFD) method designed to preserve the qualitative dynamics of the continuous system (1), particularly the non-negativity of all state variables. Let


$$X_{n}=\left(S^{n},D^{n},T_{N}^{n},T_{P}^{n},R^{n}\right)$$

represent the discrete approximation of the continuous state vector *X*(*t*_*n*_), where $t_{n}=n\Delta t$, n∈ℕ, and Δt=h>0 denotes the step size.

Motivated by the methodology introduced by Mickens [17,18], we develop a NSFD scheme that maintains the essential properties of the original model, such as positivity and boundedness of the solutions, while ensuring numerical stability. The proposed scheme is constructed to respect the nonlinearities and interactions in the system (1), offering a dynamically consistent discretization suitable for long-term simulation and analysis.


$$\frac{S^{n+1}-S^{n}}{\varphi }=\Lambda -\beta (\eta \alpha_{1}+\alpha_{2})S^{n+1}-\mu S^{n+1},$$

$$\frac{D^{n+1}-D^{n}}{\varphi }=\beta (\eta \alpha_{1}+\alpha_{2})S^{n+1}+\omega R^{n+1}+\omega_{1}T_{N}^{n+1}+\omega_{2}T_{P}^{n+1}$$

$$-(\frac{\psi T_{N}^{n}}{N^{n}}+\delta +\mu +\sigma_{1})D^{n+1},$$

$$\frac{T_{N}^{n+1}-T_{N}^{n}}{\varphi }=\frac{\psi T_{N}^{n}D^{n+1}}{N^{n}}-(\theta +\mu +\sigma_{2}+\omega_{1}+\phi )T_{N}^{n+1},$$

$$\frac{T_{P}^{n+1}-T_{P}^{n}}{\varphi }=\delta D^{n+1}-(\gamma +\mu +\sigma_{3})T_{P}^{n+1},$$

$$\frac{R^{n+1}-R^{n}}{\varphi }=\gamma T_{P}^{n+1}+\theta T_{N}^{n+1}-(\mu +\omega )R^{n+1}.$$ (2)

where the function


$$\varphi =\varphi (h)=\frac{e^{\mu h}-1}{\mu }$$

is referred to as the *nonstandard denominator function*, which satisfies $\varphi (h)=h+𝒪(h^{2})$ as h→0.

The system can also be expressed as:


$$S^{n+1}=\frac{S^{n}+\Lambda \varphi }{B_{1}},$$

$$D^{n+1}=\frac{D^{n}+\varphi (\beta (\eta \alpha_{1}+\alpha_{2})S^{n+1}+\omega R^{n+1}+\omega_{1}T_{N}^{n+1}+\omega_{2}T_{P}^{n+1})}{B_{2}},$$

$$T_{N}^{n+1}=\frac{\frac{\varphi \psi T_{N}^{n}}{N^{n}}D^{n+1}+T_{N}^{n}}{B_{3}},$$

$$T_{P}^{n+1}=\frac{\varphi \delta D^{n+1}+\varphi \phi T_{N}^{n+1}+T_{P}^{n}}{B_{4}},$$

$$R^{n+1}=\frac{\varphi \gamma T_{P}^{n+1}+\varphi \theta T_{N}^{n+1}+R^{n}}{B_{5}}.$$ (3)

Next, the NSFD scheme given in (3) is reformulated into the following explicit form:


$$S^{n+1}=\frac{\Lambda \phi +S^{n}}{B_{1}},$$

$$D^{n+1}=\frac{\varphi [\beta (\eta \alpha_{1}+\alpha_{2})S^{n+1}+\omega B_{11}+\omega_{1}B_{7}+\omega_{2}B_{9}]+D^{n}}{B_{2}-\varphi (\omega B_{10}+\omega_{1}B_{6}+\omega_{2}B_{8})},$$

$$T_{N}^{n+1}=B_{6}D^{n+1}+B_{7},$$

$$T_{P}^{n+1}=B_{8}D^{n+1}+B_{9},$$

$$R^{n+1}=B_{10}D^{n+1}+B_{11}.$$ (4)

where


$$\begin{array}{cc} B_{1}=1+\varphi (\beta (\eta \alpha_{1}+\alpha_{2})+\mu ), & B_{2}=1+\varphi (\delta +\mu +\sigma_{1})+\frac{\varphi \psi T_{N}^{n}}{N^{n}},\\ B_{3}=1+\varphi (\theta +\mu +\sigma_{2}+\omega_{1}+\phi ), & B_{4}=1+\varphi (\gamma +\mu +\sigma_{3}+\omega_{2}),\\ B_{5}=1+\varphi (\mu +\omega ),\;B_{6}=\frac{\varphi \psi T_{N}^{n}}{N^{n}B_{3}}, & B_{7}=\frac{T_{N}^{n}}{B_{3}},\;B_{8}=\frac{\varphi \delta +\varphi \phi B_{6}}{B_{4}},\\ B_{9}=\frac{\varphi \phi B_{7}+T_{P}^{n}}{B_{4}}, & B_{10}=\frac{\varphi \gamma B_{8}+\varphi \theta B_{6}}{B_{5}},\\ B_{11}=\frac{\varphi \gamma B_{9}+\varphi \theta B_{7}+R^{n}}{B_{5}}. \end{array}$$

**Theorem 3.1.** *According to [46,47], consider a nonlinear system of the form*


$$X_{t+1}=\Omega (X_{t}),$$

*where*


$$\Omega :{\mathbb{R}}^{n}\to {\mathbb{R}}^{n}$$

*is a C*^*1*^ *diffeomorphism, and let *X**_*0*_ *be an equilibrium point of the system. Then *X**_*0*_ *is locally asymptotically stable if all eigenvalues of the Jacobian matrix evaluated at the equilibrium,*


$$J(X_{0}),$$

*have absolute values less than one:*


$$|\lambda_{i}|<1,\;\text{for all }i=1,2,\ldots ,n.$$

**Proposition 3.2.** *The NSFD scheme in (4) preserves the positivity of the system variables.*

*Proof:* Suppose the initial conditions satisfy $(S^{0},D^{0},T_{N}^{0},T_{P}^{0},R^{0})\geq 0$. Since all the model parameters are positive and the denominators in the NSFD scheme remain strictly positive for any *h* > 0, it follows that $S^{n+1}\geq 0$. Consequently, this ensures that $D^{n+1}\geq 0$, $T_{N}^{n+1}\geq 0$, $T_{P}^{n+1}\geq 0$, and $R^{n+1}\geq 0$ for all *n*. Hence, the proposed NSFD scheme preserves the positivity of all state variables for any positive time step *h*. □

**Proposition 3.3.** *The NSFD method defines the discrete dynamical system in (3) within a biologically feasible region.*


$$\Omega =\left\{(S^{n},D^{n},T_{N}^{n},T_{P}^{n},R^{n}):0\leq S^{n}+D^{n}+T_{N}^{n}+T_{P}^{n}+R^{n}\leq \frac{\Lambda }{\mu }\right\}.$$

*Proof:* Let us define the total population at time step *n* as $N^{n}=S^{n}+D^{n}+T_{N}^{n}+T_{P}^{n}+R^{n}$. By summing all the equations in the system (3), we derive the following discrete relation:


$$\frac{N^{n+1}-N^{n}}{\varphi }=\Lambda -\mu N^{n}-\sigma_{1}D^{n}-\sigma_{2}T_{N}^{n}-\sigma_{3}T_{P}^{n}.$$

Solving this equation yields the bound:


$$N^{n}\leq \frac{\Lambda }{\mu }+\left(N^{0}-\frac{\Lambda }{\mu }\right)e^{-\mu nh},$$

where the initial total population is $N^{0}=S^{0}+D^{0}+T_{N}^{0}+T_{P}^{0}+R^{0}$. This result implies that $N^{n}\to \frac{\Lambda }{\mu }$ as n→∞. Therefore, the proposed NSFD scheme preserves the boundedness of the feasible region Ω for a properly chosen denominator function φ. □

### 3.2 Consistency verification

Verifying the consistency of a numerical scheme is fundamental to its reliability, as it ensures that the discrete model accurately reflects the behavior of the original continuous system. In general, this is achieved by approximating differential operators using Taylor series expansions. By truncating higher-order terms, we can obtain approximations of a desired accuracy. These omitted terms result in what is known as the truncation or discretization error. A consistent numerical scheme is one where this error vanishes as the time step and mesh size approach zero, thus aligning the numerical solution more closely with the exact solution.

Following the approach presented by [48], we apply Taylor series expansion to demonstrate the consistency of the proposed numerical method given in equation (3). Starting with the first equation of the scheme (3), we proceed as follows:


$$S^{n+1}\left\{1+\varphi (\beta (\eta \alpha_{1}+\alpha_{2})+\mu )\right\}=\Lambda \varphi +S^{n}.$$

After applying the Taylor series, we obtain


$$\left\{1+\varphi (\beta (\eta \alpha_{1}+\alpha_{2})+\mu )\right\}\left\{S^{n}+\varphi \frac{dS}{dt}+\frac{\varphi^{2}}{2!}\frac{d^{2}S}{dt}+\frac{\varphi^{3}}{3!}\frac{d^{3}S}{dt}+\cdots \right\}=S^{n}+\Lambda \varphi$$

$$S^{n}(\beta (\eta \alpha_{1}+\alpha_{2})+\mu )+\frac{dS}{dt}+\varphi \frac{dS}{dt}(\beta (\eta \alpha_{1}+\alpha_{2})+\mu )+\varphi (\frac{\varphi^{2}}{2!}\frac{d^{2}S}{dt}+\frac{\varphi^{3}}{3!}\frac{d^{3}S}{dt}+\cdots )$$

$$\left(1+\varphi (\beta (\eta \alpha_{1}+\alpha_{2})+\mu )\right)=\Lambda$$

As h→0, φ→0, we get


$$S(\beta (\eta \alpha_{1}+\alpha_{2})+\mu )+\frac{dS}{dt}=\Lambda$$

$$\frac{dS}{dt}=\Lambda -S(\beta (\eta \alpha_{1}+\alpha_{2})+\mu )$$

In a similar manner, the remaining equations of the proposed scheme (3) can be derived by applying the Taylor series expansion to each corresponding differential equation. This process ensures that all discrete approximations accurately reflect the dynamics of the original continuous system. Consequently, it can be concluded that the developed numerical method demonstrates first-order consistency, as the local truncation errors tend to zero linearly with the time step size *h*.

### 3.3 Numerical accuracy and convergence properties of the NSFD approach

In this subsection, following the approach in [15,49–52], we analyze the convergence and error bounds of the NSFD scheme given in equation (3). A numerical scheme is said to be convergent of order *p* if the global error *e*_*n*_, defined as $e_{n}:=X_{n}-X(t_{n})$ with *e*_0_ = 0, satisfies the required error bounds (see [53]).


$$e_{n}=O(l^{p}),\;\;n=1,2,\cdots ,N.$$

Let $(S(0),D(0),T_{N}(0),T_{P}(0),R(0))\in \Omega$ be any initial condition for the continuous-time model (1). Since both the continuous-time model (1) and the discrete-time model (3) are bounded, it is reasonable to define the numerical solution within the same bounded region.


$${𝒞}_{S}:=\underset{t\geq 0}{\sup}S(t),\;{𝒞}_{D}:=\underset{t\geq 0}{\sup}D(t),\;{𝒞}_{T_{N}}:=\underset{t\geq 0}{\sup}T_{N}(t),\;{𝒞}_{T_{P}}:=\underset{t\geq 0}{\sup}T_{P}(t),\;$$

$${𝒞}_{R}:=\underset{t\geq 0}{\sup}R(t),{𝒟}_{S}:=\underset{n\geq 0}{\sup}\{S^{n}\},\;{𝒟}_{D}:=\underset{n\geq 0}{\sup}\{D^{n}\},\;{𝒟}_{T_{N}}:=\underset{n\geq 0}{\sup}\{T_{N}^{n}\},\;{𝒟}_{T_{P}}:=\underset{n\geq 0}{\sup}\{T_{P}^{n}\},$$

$${𝒟}_{R}:=\underset{n\geq 0}{\sup}\{R^{n}\},\;\Omega^{CD}:=\{(S,D,T_{N},T_{P},R)|0\leq S\leq \max\{{𝒞}_{S},{𝒟}_{S}\},\;0\leq D\leq \max\{{𝒞}_{D},{𝒟}_{D}\},\;$$

$$.0\leq T_{N}\leq \max\{{𝒞}_{T_{N}},{𝒟}_{T_{N}}\},\;0\leq T_{P}\leq \max\{{𝒞}_{T_{P}},{𝒟}_{T_{P}}\},\;0\leq R\leq \max\{{𝒞}_{R},{𝒟}_{R}\}\}\;$$

For each arbitrary but fixed step size *h*  > 0, we denote


$$\Omega^{𝒟}:=\left\{(S,D,T_{N},T_{P},R)|0\leq S\leq {𝒟}_{S},\;0\leq D\leq {𝒟}_{D},\;0\leq T_{N}\leq {𝒟}_{T_{N}},\;0\leq T_{P}\leq {𝒟}_{T_{P}},\;0\leq R\leq {𝒟}_{R},\;0\leq h\leq h^{*}\right\}.$$

We denote the right-hand side functions of system (1) as $f_{1}(S,D,T_{N},T_{P},R),\;f_{2}(S,D,T_{N},T_{P},R),\;f_{3}(S,D,T_{N},T_{P},R),$ $f_{4}(S,D,T_{N},T_{P},R),$ $f_{5}(S,D,T_{N},T_{P},R),$ corresponding to each of the five equations, respectively.

Due to the boundedness of the state variables $S,D,T_{N},T_{P},R$, and the continuity of the functions $f_{1},f_{2},f_{3},f_{4},f_{5}$, we can define


$${ℳ}_{1}^{**}:=\underset{t\geq 0}{\sup}|S^{\prime \prime }(t)|,\;{ℳ}_{2}^{**}:=\underset{t\geq 0}{\sup}|D^{\prime \prime }(t)|,\;{ℳ}_{3}^{**}:=\underset{t\geq 0}{\sup}|{T_{N}^{\prime }}^{\prime }(t)|,\;{ℳ}_{4}^{**}:=\underset{t\geq 0}{\sup}|{T_{P}^{\prime }}^{\prime }(t)|,$$

$${ℳ}_{5}^{**}:=\underset{t\geq 0}{\sup}|R^{\prime \prime }(t)|,$$

$${ℳ}^{**}:={ℳ}_{1}^{**}+{ℳ}_{2}^{**}+{ℳ}_{3}^{**}+{ℳ}_{4}^{**}+{ℳ}_{5}^{**},$$ (5)

and


$$m_{l}^{S}:=\max_{(S,D,T_{N},T_{P},R)\in \Omega^{CD}}\left|\frac{\partial f_{l}}{\partial S}(S,D,T_{N},T_{P},R)\right|,\;\;l=1,2,3,4,5,$$

$$m_{l}^{D}:=\max_{(S,D,T_{N},T_{P},R)\in \Omega^{CD}}\left|\frac{\partial f_{l}}{\partial D}(S,D,T_{N},T_{P},R)\right|,\;\;l=1,2,3,4,5,$$

$$m_{l}^{T_{N}}:=\max_{(S,D,T_{N},T_{P},R)\in \Omega^{CD}}\left|\frac{\partial f_{l}}{\partial T_{N}}(S,D,T_{N},T_{P},R)\right|,\;\;l=1,2,3,4,5,$$ (6)

$$m_{l}^{T_{P}}:=\max_{(S,D,T_{N},T_{P},R)\in \Omega^{CD}}\left|\frac{\partial f_{l}}{\partial T_{P}}(S,D,T_{N},T_{P},R)\right|,\;\;l=1,2,3,4,5,$$

$$m_{l}^{R}:=\max_{(S,D,T_{N},T_{P},R)\in \Omega^{CD}}\left|\frac{\partial f_{l}}{\partial R}(S,D,T_{N},T_{P},R)\right|,\;\;l=1,2,3,4,5,$$

$$m^{*}=\max\left\{\sum_{l=1}^{5}m_{l}^{S},\;\sum_{l=1}^{5}m_{l}^{D},\;\sum_{l=1}^{5}m_{l}^{T_{N}},\;\sum_{l=1}^{5}m_{l}^{T_{P}},\;\sum_{l=1}^{5}m_{l}^{R}\right\}.$$

Likewise, we can represent the right-hand side of all the eqution of NSFD model (4) as $g_{1}(S^{n},D^{n},T_{N}^{n},T_{P}^{n},R^{n},h),$ $g_{2}(S^{n},D^{n},T_{N}^{n},T_{P}^{n},R^{n},h),\;g_{3}(S^{n},D^{n},T_{N}^{n},T_{P}^{n},R^{n},h),$ $\;g_{4}(S^{n},D^{n},T_{N}^{n},T_{P}^{n},R^{n},h),g_{5}(S^{n},D^{n},T_{N}^{n},T_{P}^{n},R^{n},h),$ respectively. It is straightforward to verify that


$$g_{1}(S^{n},D^{n},T_{N}^{n},T_{P}^{n},R^{n},0)\equiv S^{n},\;\frac{\partial g_{1}}{\partial h}(S^{n},D^{n},T_{N}^{n},T_{P}^{n},R^{n},0)\equiv f_{1}(S^{n},D^{n},T_{N}^{n},T_{P}^{n},R^{n},0),$$

$$g_{2}(S^{n},D^{n},T_{N}^{n},T_{P}^{n},R^{n},0)\equiv D^{n},\;\frac{\partial g_{2}}{\partial h}(S^{n},D^{n},T_{N}^{n},T_{P}^{n},R^{n},0)\equiv f_{2}(S^{n},D^{n},T_{N}^{n},T_{P}^{n},R^{n},0),$$

$$g_{3}(S^{n},D^{n},T_{N}^{n},T_{P}^{n},R^{n},0)\equiv T_{N}^{n},\;\frac{\partial g_{3}}{\partial h}(S^{n},D^{n},T_{N}^{n},T_{P}^{n},R^{n},0)\equiv f_{3}(S^{n},D^{n},T_{N}^{n},T_{P}^{n},R^{n},0),$$

$$g_{4}(S^{n},D^{n},T_{N}^{n},T_{P}^{n},R^{n},0)\equiv T_{P}^{n},\;\frac{\partial g_{4}}{\partial h}(S^{n},D^{n},T_{N}^{n},T_{P}^{n},R^{n},0)\equiv f_{4}(S^{n},D^{n},T_{N}^{n},T_{P}^{n},R^{n},0),$$

$$g_{5}(S^{n},D^{n},T_{N}^{n},T_{P}^{n},R^{n},0)\equiv R^{n},\;\frac{\partial g_{5}}{\partial h}(S^{n},D^{n},T_{N}^{n},T_{P}^{n},R^{n},0)\equiv f_{5}(S^{n},D^{n},T_{N}^{n},T_{P}^{n},R^{n},0).\;$$ (7)

Assume that $\phi^{\prime \prime }(h)$ exists and is bounded for all *h* > 0. Let us define


$${ℒ}_{1}^{**}=\max_{(S^{n},D^{n},T_{N}^{n},T_{P}^{n},R^{n},h)\in \Omega^{𝒟}}\left|\frac{\partial^{2}g_{1}}{\partial h^{2}}(S^{n},D^{n},T_{N}^{n},T_{P}^{n},R^{n},h)\right|,$$

$${ℒ}_{2}^{**}=\max_{(S^{n},D^{n},T_{N}^{n},T_{P}^{n},R^{n},h)\in \Omega^{𝒟}}\left|\frac{\partial^{2}g_{2}}{\partial h^{2}}(S^{n},D^{n},T_{N}^{n},T_{P}^{n},R^{n},h)\right|,$$

$${ℒ}_{3}^{**}=\max_{(S^{n},D^{n},T_{N}^{n},T_{P}^{n},R^{n},h)\in \Omega^{𝒟}}\left|\frac{\partial^{2}g_{3}}{\partial h^{2}}(S^{n},D^{n},T_{N}^{n},T_{P}^{n},R^{n},h)\right|,$$

$${ℒ}_{4}^{**}=\max_{(S^{n},D^{n},T_{N}^{n},T_{P}^{n},R^{n},h)\in \Omega^{𝒟}}\left|\frac{\partial^{2}g_{4}}{\partial h^{2}}(S^{n},D^{n},T_{N}^{n},T_{P}^{n},R^{n},h)\right|,$$

$${ℒ}_{5}^{**}=\max_{(S^{n},D^{n},T_{N}^{n},T_{P}^{n},R^{n},h)\in \Omega^{𝒟}}\left|\frac{\partial^{2}g_{5}}{\partial h^{2}}(S^{n},D^{n},T_{N}^{n},T_{P}^{n},R^{n},h)\right|,$$ (8)

$${ℒ}^{**}={ℒ}_{1}^{**}+{ℒ}_{2}^{**}+{ℒ}_{3}^{**}+{ℒ}_{4}^{**}+{ℒ}_{5}^{**}.$$

The constructed NSFD scheme (4) is shown to be first-order convergent. Furthermore, the error can be estimated by the following bound:

$$\left|S(t_{n})-S^{n}|+|D(t_{n})-D^{n}|+|T_{N}(t_{n})-T_{N}^{n}|\;\;+|T_{P}(t_{n})-T_{P}^{n}|+|R(t_{n})-R^{n}|\leq \frac{h\left({ℒ}^{**}+{ℳ}^{**}\right)}{2m^{*}}(e^{m^{*}t_{n}}-1\right)$$ (9)

for all n≥0, where the constants *m* , ${ℳ}^{**}$, and ${ℒ}^{**}$ are defined in equations (6), (5), and (8), respectively.

*Proof:* Applying Taylor’s theorem, we obtain


$$S(t_{n}+1)=S(t_{n})+hf_{1}(S(t_{n}),D(t_{n}),T_{N}(t_{n}),T_{P}(t_{n}),R(t_{n}))+\frac{h^{2}}{2}S^{\prime \prime }(\nu S),\;t_{n}<\nu S<t_{n+1},$$

$$D(t_{n}+1)=D(t_{n})+hf_{2}(S(t_{n}),D(t_{n}),T_{N}(t_{n}),T_{P}(t_{n}),R(t_{n}))+\frac{h^{2}}{2}D^{\prime \prime }(\nu D),\;t_{n}<\nu D<t_{n+1},$$

$$T_{N}(t_{n}+1)=T_{N}(t_{n})+hf_{3}(S(t_{n}),D(t_{n}),T_{N}(t_{n}),T_{P}(t_{n}),R(t_{n}))+\frac{h^{2}}{2}{T_{N}^{\prime }}^{\prime }(\nu T_{N}),\;t_{n}<\nu T_{N}<t_{n+1},$$

$$T_{P}(t_{n}+1)=T_{P}(t_{n})+hf_{4}(S(t_{n}),D(t_{n}),T_{N}(t_{n}),T_{P}(t_{n}),R(t_{n}))+\frac{h^{2}}{2}{T_{P}^{\prime }}^{\prime }(\nu T_{P}),\;t_{n}<\nu T_{P}<t_{n+1},$$

$$R(t_{n}+1)=R(t_{n})+hf_{5}(S(t_{n}),D(t_{n}),T_{N}(t_{n}),T_{P}(t_{n}),R(t_{n}))+\frac{h^{2}}{2}R^{\prime \prime }(\nu R),\;t_{n}<\nu R<t_{n+1}.$$ (10)

As a consequence of Taylor’s theorem and equation (7), we obtain the following :


$$S^{n+1}=S^{n}+hf_{1}(S^{n},D^{n},T_{N}^{n},T_{P}^{n},R^{n})+\frac{h^{2}}{2}\frac{\partial^{2}g_{1}}{\partial h^{2}}(S^{n},D^{n},T_{N}^{n},T_{P}^{n},R^{n},h_{S}),\;0<h_{S}<h,$$

$$D^{n+1}=D^{n}+hf_{2}(S^{n},D^{n},T_{N}^{n},T_{P}^{n},R^{n})+\frac{h^{2}}{2}\frac{\partial^{2}g_{2}}{\partial h^{2}}(S^{n},D^{n},T_{N}^{n},T_{P}^{n},R^{n},h_{D}),\;0<h_{D}<h,$$

$$T_{N}^{n+1}=T_{N}^{n}+hf_{3}(S^{n},D^{n},T_{N}^{n},T_{P}^{n},R^{n})+\frac{h^{2}}{2}\frac{\partial^{2}g_{3}}{\partial h^{2}}(S^{n},D^{n},T_{N}^{n},T_{P}^{n},R^{n},h_{T_{N}}),\;0<h_{T_{N}}<h,$$

$$T_{P}^{n+1}=T_{P}^{n}+hf_{4}(S^{n},D^{n},T_{N}^{n},T_{P}^{n},R^{n})+\frac{h^{2}}{2}\frac{\partial^{2}g_{4}}{\partial h^{2}}(S^{n},D^{n},T_{N}^{n},T_{P}^{n},R^{n},h_{S}),\;0<h_{T_{P}}<h,$$

$$R^{n+1}=R^{n}+hf_{5}(S^{n},D^{n},T_{N}^{n},T_{P}^{n},R^{n})+\frac{h^{2}}{2}\frac{\partial^{2}g_{5}}{\partial h^{2}}(S^{n},D^{n},T_{N}^{n},T_{P}^{n},R^{n},h_{R}),\;0<h_{R}<h.$$ (11)

Next,


$$\left|f_{j}(S(t_{n}),\;D(t_{n}),\;T_{N}(t_{n}),\;T_{P}(t_{n}),\;R(t_{n}))-f_{j}(S^{n},\;D^{n},\;T_{N}^{n},\;T_{P}^{n},\;R^{n})\right|$$

$$=|\frac{\partial f_{j}}{\partial S}(\Phi_{j})(S(t_{n})-S^{n})+\frac{\partial f_{j}}{\partial D}(\Phi_{j})(D(t_{n})-D^{n})+\frac{\partial f_{j}}{\partial T_{N}}(\Phi_{j})(T_{N}(t_{n})-T_{N}^{n})\;\;+\frac{\partial f_{j}}{\partial T_{P}}(\Phi_{j})(T_{P}(t_{n})-T_{P}^{n})+\frac{\partial f_{j}}{\partial R}(\Phi_{j})(R(t_{n})-R^{n})+|$$

$$\leq \left|\frac{\partial f_{j}}{\partial S}(\Phi_{j})\right||(S(t_{n})-S^{n})|+\left|\frac{\partial f_{j}}{\partial D}(\Phi_{j})\right||(D(t_{n})-D^{n})|+\left|\frac{\partial f_{j}}{\partial T_{N}}(\Phi_{j})\right||(T_{N}(t_{n})-T_{N}^{n})|\;\;+\left|\frac{\partial f_{j}}{\partial T_{P}}(\Phi_{j})\right||(T_{P}(t_{n})-T_{P}^{n})|+\left|\frac{\partial f_{j}}{\partial R}(\Phi_{j})\right||(R(t_{n})-R^{n})|$$

$$\leq m_{j}^{S}|(S(t_{n})-S^{n})|+m_{j}^{D}|(D(t_{n})-D^{n})|+m_{j}^{T_{N}}|(T_{N}(t_{n})-T_{N}^{n})|+m_{j}^{T_{P}}|(T_{P}(t_{n})-T_{P}^{n})|\;\;+m_{j}^{R}|(R(t_{n})-R^{n})|,\;\;j=1,2,3,4,5$$ (12)

where $m_{j}^{S},\;m_{j}^{D},\;m_{j}^{T_{N}},\;m_{j}^{T_{P}},\;m_{j}^{R}$ for j=1,2,3,4,5 are defined in equation (6), and $\Phi_{j}$ (for j=1,2,3,4,5) denote points lying between $\left(S(t_{n}),D(t_{n}),T_{N}(t_{n}),T_{P}(t_{n}),R(t_{n})\right)$ and $\left(S^{n},D^{n},T_{N}^{n},T_{P}^{n},R^{n}\right)$. Let us denote for each n≥0


$$e_{n}^{S}:=S(t_{n})-S^{n},\;e_{n}^{D}:=D(t_{n})-D^{n},\;e_{n}^{T_{N}}:=T_{N}(t_{n})-T_{N}^{n},\;e_{n}^{T_{P}}:=T_{P}(t_{n})-T_{P}^{n},\;\;e_{n}^{R}:=R(t_{n})-R^{n},\;e_{n}:=|e_{n}^{S}|+|e_{n}^{D}|+|e_{n}^{T_{N}}|+|e_{n}^{T_{P}}|+|e_{n}^{R}|.$$

It follows from (10), (11), (12), that


$$|e_{n+1}^{S}|=|e_{n}^{S}+h(f_{1}(S(t_{n}),D(t_{n}),T_{N}(t_{n}),T_{P}(t_{n}),R(t_{n}))-f_{1}(S^{n},D^{n},T_{N}^{n},T_{P}^{n},R^{n}))\;\;+\frac{h^{2}}{2}S^{\prime \prime }(\nu S)-\frac{h^{2}}{2}\frac{\partial^{2}g_{1}}{\partial h^{2}}(S^{n},D^{n},T_{N}^{n},T_{P}^{n},R^{n},h_{s})|\;\leq |e_{n}^{s}|+h\left(m_{1}^{S}|e_{n}^{S}|+m_{1}^{D}|e_{n}^{D}|+m_{1}^{T_{N}}|e_{n}^{T_{N}}|+m_{1}^{T_{P}}|e_{n}^{T_{P}}|+m_{1}^{R}|e_{n}^{R}|\right)\;\;+\frac{h^{2}}{2}\left({ℳ}_{1}^{**}+{ℒ}_{1}^{**}\right).$$

Similarly,


$$|e_{n+1}^{D}|\leq |e_{n}^{D}|+h\left(m_{2}^{S}|e_{n}^{S}|+m_{2}^{D}|e_{n}^{D}|+m_{2}^{T_{N}}|e_{n}^{T_{N}}|+m_{2}^{T_{P}}|e_{n}^{T_{P}}|+m_{2}^{R}|e_{n}^{R}|\right)+\frac{h^{2}}{2}\left({ℳ}_{2}^{**}+{ℒ}_{2}^{**}\right).$$

$$|e_{n+1}^{T_{N}}|\leq |e_{n}^{T_{N}}|+h\left(m_{3}^{S}|e_{n}^{S}|+m_{3}^{D}|e_{n}^{D}|+m_{3}^{T_{N}}|e_{n}^{T_{N}}|+m_{3}^{T_{P}}|e_{n}^{T_{P}}|+m_{3}^{R}|e_{n}^{R}|\right)+\frac{h^{2}}{2}\left({ℳ}_{3}^{**}+{ℒ}_{3}^{**}\right).$$

$$|e_{n+1}^{T_{P}}|\leq |e_{n}^{T_{P}}|+h\left(m_{4}^{S}|e_{n}^{S}|+m_{4}^{D}|e_{n}^{D}|+m_{4}^{T_{N}}|e_{n}^{T_{N}}|+m_{4}^{T_{P}}|e_{n}^{T_{P}}|+m_{4}^{R}|e_{n}^{R}|\right)+\frac{h^{2}}{2}\left({ℳ}_{4}^{**}+{ℒ}_{4}^{**}\right).$$

$$|e_{n+1}^{R}|\leq |e_{n}^{R}|+h\left(m_{5}^{S}|e_{n}^{S}|+m_{5}^{D}|e_{n}^{D}|+m_{5}^{T_{N}}|e_{n}^{T_{N}}|+m_{5}^{T_{P}}|e_{n}^{T_{P}}|+m_{5}^{R}|e_{n}^{R}|\right)+\frac{h^{2}}{2}\left({ℳ}_{5}^{**}+{ℒ}_{5}^{**}\right).$$

Hence,


$$\left|e_{n+1}^{S}\right|+\left|e_{n+1}^{D}\right|+\left|e_{n+1}^{T_{N}}\right|+\left|e_{n+1}^{T_{P}}\right|+\left|e_{n+1}^{R}\right|\;\leq \left(\left|e_{n}^{S}\right|+\left|e_{n}^{D}\right|+\left|e_{n}^{T_{N}}\right|+\left|e_{n}^{T_{P}}\right|+\left|e_{n}^{R}\right|\right)+\frac{h^{2}}{2}\left({ℳ}^{**}+{ℒ}^{**}\right)\;\;+h\left(\sum_{j=1}^{5}m_{j}^{S}\left|e_{n}^{S}\right|+\sum_{j=1}^{5}m_{j}^{D}\left|e_{n}^{D}\right|+\sum_{j=1}^{5}m_{j}^{T_{N}}\left|e_{n}^{T_{N}}\right|+\sum_{j=1}^{5}m_{j}^{T_{P}}\left|e_{n}^{T_{P}}\right|+\sum_{j=1}^{5}m_{j}^{R}\left|e_{n}^{R}\right|\right)+\;\leq (1+m^{*}h)\left(\left|e_{n}^{S}\right|+\left|e_{n}^{D}\right|+\left|e_{n}^{T_{N}}\right|+\left|e_{n}^{T_{P}}\right|+\left|e_{n}^{R}\right|\right)+\frac{h^{2}}{2}\left({ℳ}^{**}+{ℒ}^{**}\right)$$

Therefore, we have that:

$$e_{n+1}\leq (1+m^{*}h)e_{n}+\frac{h^{2}\Psi }{2},\;\;\Psi ={ℳ}^{**}+{ℒ}^{**}$$ (13)

where $m^{*},\;{ℳ}^{**},\;{ℒ}^{**}$ are defined in (6), (5), and (8), respectively. It follows from (13) and the initial condition *e*_0_ = 0 that the result holds. that


$$e_{n+1}\leq (1+m^{*}h)e_{n}+\frac{h^{2}\Psi }{2},\;\leq (1+m^{*}h)\left[(1+m^{*}h)e_{n+1}+\frac{h^{2}\Psi }{2}\right]+\frac{h^{2}\Psi }{2}\;=\;(1+m^{*}h)e_{n+1}+\frac{h^{2}\Psi }{2}\;\;\left[1+(1+m^{*}h)\right]\leq \cdots \leq (1+m^{*}h{)}^{n}e_{0}+\frac{h^{2}\Psi }{2}\sum_{j=0}^{n}(1+m^{*}h{)}^{j}\;\leq \frac{h\Psi }{2m*}\left[(1+m^{*}h{)}^{n+1}-1\right]$$

Applying the well-known inequality $1+d\leq e^{d}$ for d≥0, we deduce the following result.


$$e_{n+1}\leq \frac{h\Psi }{2m^{*}}\left[e^{m^{*}h(n+1)}-\right]\;=\;\frac{h\Psi }{2m^{*}}\left[1+m^{*t_{n+1}}-1\right]$$

This establishes the desired result. Hence, the proof is complete. □

### 3.4 Numerical validation

This section presents numerical experiments to validate the performance of the NSFD method in comparison with Euler and RK4 schemes. Simulations were performed using the parameter values in Table 1 for various step sizes h=0.001,0.05,0.5,1.00,1.25,1.65,2.00,2.5. For small step sizes (h≤1.25), all three methods yield stable solutions. However, for larger step sizes (*h* > 1.25), the Euler and RK4 methods become unstable and diverge, whereas the NSFD method continues to produce biologically consistent and bounded results.

### 3.5 Stability and convergence analysis

Tables 2 and 3 provide insight into the numerical behavior of each method. The spectral radius of the Jacobian matrix (Table 2) confirms that the NSFD scheme remains stable with radius less than one across all tested step sizes. In contrast, the Euler and RK4 methods exhibit spectral radii greater than one for larger step sizes, indicating instability. The convergence summary (Table 3) further highlights that only NSFD maintains convergence for all step sizes tested, while RK4 and Euler diverge for h≥1.65 and h≥1.25, respectively.

**Table 2:** Spectral radius *ρ* for different step sizes and numerical schemes.

Method\Step size *h*	0.001	0.05	0.5	1.00	1.25	1.65	2.00	2.5
NSFD	0.9999	0.9974	0.9845	0.9746	0.9674	0.9525	0.9378	0.9168
Euler	0.9993	0.9972	0.9724	0.9448	0.9310	1.4083	1.9192	2.649077
RK4	0.9994	0.9973	0.9727	0.9463	0.9334	0.9129	1.2214	3.2983

**Table 3:** Convergence behavior (C: Convergent, D: Divergent) of each method for different step sizes.

Method\Step size *h*	0.001	0.05	0.5	1.00	1.25	1.65	2.00	2.5
NSFD	C	C	C	C	C	C	C	C
Euler	C	C	C	C	C	D	D	D
RK4	C	C	C	C	C	C	D	D

### 3.6 Simulation results

**Fig 13:**
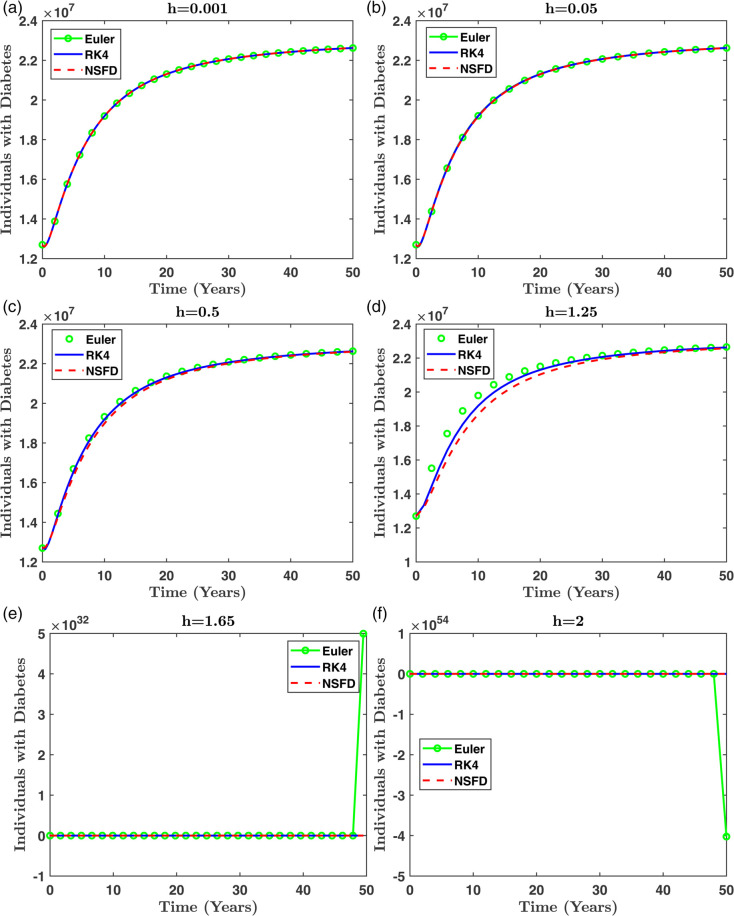
Comparison of different numerical techniques for various step sizes (h) (a) h=0.001, (b) h=0.05, (c) h=0.5, (d) h=1.25, (e) h=1.65 and (f) h=2 in simulating the dynamics of individuals with diabetes.

**Fig 14:**
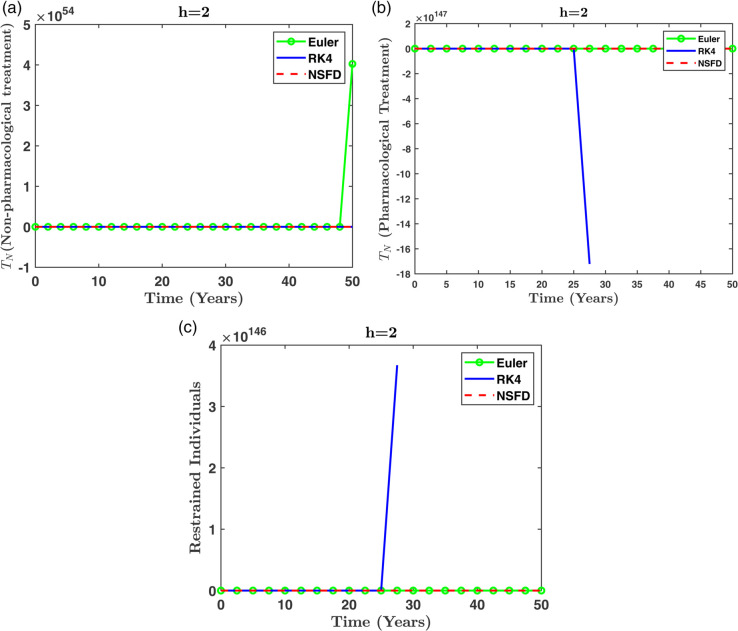
Comparison of different numerical techniques for various step sizes (h) in simulating the dynamics of individuals: (a) under non-pharmacological treatment, (b) under pharmacological treatment, and (c) restrained individuals.

Fig 13 shows the dynamics of the diabetic population *D*(*t*) for different methods across step sizes. The NSFD method closely follows the expected trajectory, while divergence is clearly observed in RK4 and Euler for larger *h*. Fig 14 presents the evolution of *T*_*N*_(*t*), *T*_*P*_(*t*), and *R*(*t*) at *h* = 2, reinforcing the superior stability of NSFD under coarse discretization.To reduce redundancy, only a subset of figures is included in the manuscript. Selected compartments at *h* = 2 are shown to represent the comparative performance of all methods.

**Fig 15:**
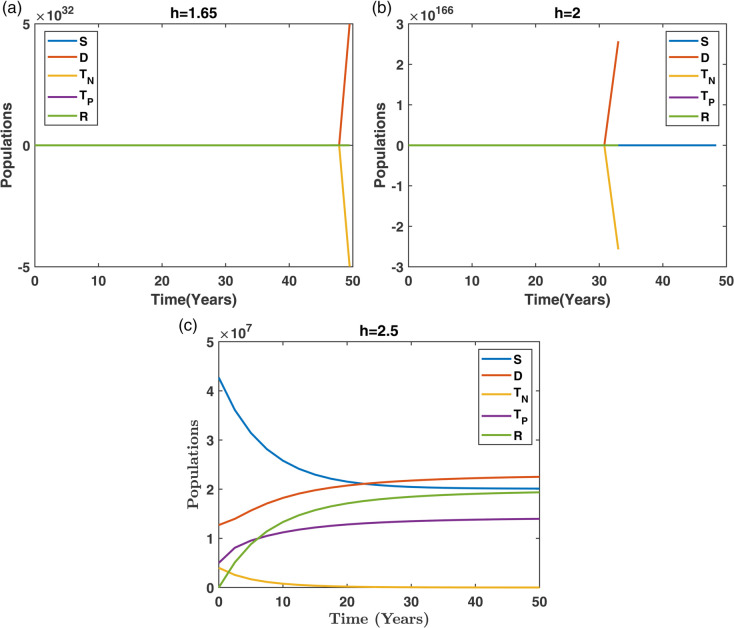
Comparison of NSFD techniques for various step sizes (h): (a) h=1.65, (b) h=2, and (c) h=2.5 in simulating the dynamics of the entire population.

Fig 15 displays the combined population dynamics for all compartments. The NSFD scheme remains stable across all step sizes, while RK4 and Euler produce unrealistic and unstable solutions for larger *h*, confirming NSFD’s robustness.

### 3.7 Error and convergence analysis of Forward Euler, RK4, and NSFD methods

In this section, we analyze the numerical behavior of model (4) over the time interval [0,50], using the parameter values listed in Table 1. Since an exact analytical solution is not available, we consider the numerical solution obtained using the fourth-order Runge–Kutta (RK4) method with a very small step size of *h* = 10^−3^ as the reference solution.

Table 4 presents the absolute errors and estimated convergence rates for the Euler method, RK4 method, and Nonstandard Finite Difference (NSFD) method, calculated at the final simulation time. The total absolute error is defined as:

**Table 4:** Comparison of error and convergence rates for Euler, RK4, and NSFD methods.

Step Size	Euler Error	Euler Rate	RK4 Error	RK4 Rate	NSFD Error	NSFD Rate
1.25	6.1749e-02	—	8.4925e-03	—	2.6604e-02	—
1.00	4.2564e-02	1.6679	2.87718e-03	4.8589	2.1541e-02	0.9456
0.75	2.5934e-02	1.7224	7.0450e-04	4.8848	1.6379e-02	0.9519
0.50	1.2690e-02	1.7624	9.6278e-05	4.9085	1.1055e-02	0.9686
0.05	1.2349e-03	1.0118	5.8570e-09	4.2159	1.1309e-03	0.9903
0.005	1.2096e-04	1.0089	5.5707e-13	4.0217	1.1132e-04	1.0070
0.001	2.2212e-05	1.0533	3.8730e-13	0.2259	2.0450e-05	1.0527


$$\text{Error}=|S(t_{N})-S^{N}|+|D(t_{N})-D^{N}|+|T_{N}(t_{N})-T_{N}^{N}|+|T_{P}(t_{N})-T_{P}^{N}|+|R(t_{N})-R^{N}|,$$

and the corresponding convergence rate is computed using the standard formula (see [53]):


$$\text{Rate}=\frac{\log\left(\frac{h_{1}}{h_{2}}\right)}{\log\left(\frac{\text{Error}(h_{1})}{\text{Error}(h_{2})}\right)}.$$

From Table 4, we observe that both the Euler and NSFD methods exhibit convergence rates close to one, confirming their first-order accuracy. This is consistent with their theoretical properties and validates Theorem 3.3 of [53] for the NSFD scheme. On the other hand, the RK4 method shows a significantly higher rate initially, consistent with its expected fourth-order accuracy. However, a slight decline in convergence rate is observed for finer step sizes, likely due to numerical round-off and cumulative error effects phenomena similarly noted in Example 4.1 of [53].

The numerical investigation demonstrates that the NSFD method outperforms classical schemes by preserving stability across a wide range of step sizes, maintaining convergence even under coarse time discretization, and capturing biologically meaningful long-term dynamics. Thus, the NSFD scheme serves as a reliable and efficient approach for simulating chronic disease dynamics, particularly in scenarios where standard methods fail due to numerical instability.

## 4 Optimal control

Optimal control analysis plays a vital role in various fields, particularly in the modeling and management of chronic diseases like diabetes. It enables researchers to design and evaluate strategies aimed at minimizing the burden of the disease and enhancing the effectiveness of intervention measures. In this study, we develop and analyze a mathematical model for diabetes to assess the impact of different control strategies such as prevention, early diagnosis, lifestyle modifications, and treatment adherence on the disease dynamics. The insights gained from this analysis can provide valuable guidance for public health decision-makers in formulating effective policies and interventions for managing and controlling diabetes.

Given this, we incorporate three control parameters into the Diabetes model, which is represented by the system (1), which are:

1. *u*_1_: This control focuses on raising awareness to reduce the risk of developing diabetes. It includes public health education campaigns, lifestyle counseling, and dissemination of information about risk factors such as unhealthy diet, lack of physical activity, and obesity. Implementing effective *u*_1_ strategies can encourage behavioral changes that help prevent the onset of diabetes.
2. *u*_2_: The control *u*_2_ targets the promotion of non-pharmacological treatments in the management of diabetes. These include regular physical activity, dietary modifications, weight management, and other lifestyle-based interventions that are essential for maintaining glycemic control and preventing disease progression. The treatment rate associated with these non-pharmacological efforts is represented by (ψ  +  $\tau u_{2})$, where *ψ* denotes the baseline treatment rate in the absence of control, and *τ* is a positive parameter that measures the effectiveness of the control strategy. Specifically, *τ* quantifies how much the treatment rate improves in response to the implementation of *u*_2_. A higher value of *τ* indicates that even small efforts in promoting lifestyle changes can lead to substantial improvements in health outcomes. Therefore, enhancing *u*_2_ can play a crucial role in delaying disease progression, improving patient outcomes, and reducing the dependency on pharmacological interventions during the early or pre-diabetic stages.
3. *u*_3_: The control *u*_3_ is introduced to enhance pharmacological treatment for individuals with diabetes. This includes improving access to essential medications such as insulin and oral hypoglycemic agents, promoting adherence to prescribed treatment regimens, and ensuring consistent medical follow-up. The treatment rate associated with pharmacological intervention is modeled as *δ*  +  $\rho u_{3}$, where *δ* denotes the baseline pharmacological treatment rate, and *ρ* is a positive parameter that reflects the effectiveness of the control *u*_3_. In this context, *ρ* captures the degree to which the pharmacological treatment rate increases in response to the intensity of the control effort. A higher value of *ρ* indicates that greater improvement in treatment outcomes can be achieved even with moderate investment in pharmacological interventions. By optimizing *u*_3_, the model aims to reduce diabetes-related complications, improve long-term health outcomes, and lower healthcare costs associated with disease progression.

The pre-fixed period over which the three controls are applied, denoted by *t*_*p*_, is bounded, and each control function $u_{1},u_{2},u_{3}$ is assumed to be Lebesgue integrable on *[*0,*t*_*p*_*]*. When a control function equals zero, it indicates that no effort is applied for that specific intervention, while a value of one represents the maximum possible effort. Accordingly, each control is taken to lie within the interval [0,1]. For clarity, these three controls have been incorporated into the model (1).


$$\frac{dS}{dt}=\Lambda -(1-u_{1}(t))\beta (\eta \alpha_{1}+\alpha_{2})S-\mu S,$$

$$\frac{dD}{dt}=(1-u_{1}(t))\beta (\eta \alpha_{1}+\alpha_{2})S+\omega R+\omega_{1}T_{N}+\omega_{2}T_{P}\;\;-\frac{(\psi +\tau u_{2}(t))T_{N}D}{N}-(\delta +\rho u_{3}(t))D-(\mu +\sigma_{1})D,$$

$$\frac{dT_{N}}{dt}=\frac{(\psi +\tau u_{2}(t))T_{N}D}{N}-(\theta +\mu +\sigma_{2}+\omega_{1}+\phi )T_{N},$$

$$\frac{dT_{P}}{dt}=(\delta +\rho u_{3}(t))D+\phi T_{N}-(\gamma +\mu +\sigma_{3}+\omega_{2})T_{P},$$

$$\frac{dR}{dt}=\gamma T_{P}+\theta T_{N}-(\mu +\omega )R$$ (14)

For fixed *t*_*p*_, the objective functional is provided by


$$J=\int_{0}^{t_{p}}\left(C_{1}D+C_{2}T_{N}+C_{3}T_{P}+\frac{1}{2}C_{4}u_{1}^{2}+\frac{1}{2}C_{5}u_{2}^{2}+\frac{1}{2}C_{6}u_{3}^{2}\right)dt,$$

where $C_{1},C_{2},C_{3},C_{4},C_{5},C_{6}\geq 0$ are the weight constants.

The goal is to determine the control parameters $u_{1}^{⋆},u_{2}^{⋆},u_{3}^{⋆}$ such that


$$J(u_{1}^{⋆},u_{2}^{⋆},u_{3}^{⋆})=\min_{u_{1},u_{2},u_{3}\in \Gamma }J(u_{1},u_{2},u_{3}),$$

where Γ is the control set, defined as


$$\Gamma =\{u_{1},u_{2},u_{3}:\;\text{measurable and}\;\;0\leq u_{1},u_{2},u_{3}<1\}\;\;\text{and}\;\;t\in [0,t_{p}]$$

The Lagrangian of this problem is:


$$L(D,T_{N},T_{P},u_{1},u_{2},u_{3})=C_{1}D+C_{2}T_{N}+C_{3}T_{P}+\frac{1}{2}C_{4}u_{1}^{2}+\frac{1}{2}C_{5}u_{2}^{2}+\frac{1}{2}C_{6}u_{3}^{2}.$$

The Hamiltonian $H_{C}$ formed for our problem is:

$$H_{C}=L(D,T_{N},T_{P},u_{1},u_{2},u_{3})+\kappa_{1}\frac{dS}{dt}+\kappa_{2}\frac{dD}{dt}+\kappa_{3}\frac{dT_{N}}{dt}+\kappa_{4}\frac{dT_{P}}{dt}+\kappa_{5}\frac{dR}{dt}.$$ (15)

where $\kappa_{i}^{\prime }s$ are the adjoint variables (i=1 to 5). Following are the differential equations that represent the adjoint variables.


$$\frac{d\kappa_{1}}{dt}=-\frac{\partial H_{C}}{\partial S}=(1-u_{1}(t))\beta (\eta \alpha_{1}+\alpha_{2})(\kappa_{1}-\kappa_{2})+(\psi +\tau u_{2}(t))\frac{T_{N}D}{N^{2}}(\kappa_{3}-\kappa_{2})+\mu \kappa_{1}$$

$$\frac{d\kappa_{2}}{dt}=-\frac{\partial H_{C}}{\partial D}=(\psi +\tau u_{2}(t))\left(\frac{T_{N}(N-D)}{N^{2}}\right)(\kappa_{2}-\kappa_{3})+(\delta +\rho u_{3}(t))(\kappa_{2}-\kappa_{4})+\kappa_{2}(\mu +\sigma_{1})-C_{1}$$

$$\frac{d\kappa_{3}}{dt}=-\frac{\partial H_{C}}{\partial T_{N}}=\omega_{1}(\kappa_{3}-\kappa_{2})+\phi (\kappa_{3}-\kappa_{4})+(\psi +\tau u_{2}(t))\left(\frac{D(N-T_{N})}{N^{2}}\right)(\kappa_{2}-\kappa_{3})\;\;+\theta (\kappa_{3}-\kappa_{5})+(\mu +\sigma_{2})\kappa_{3}-C_{2}$$

$$\frac{d\kappa_{4}}{dt}=-\frac{\partial H_{C}}{\partial T_{P}}=(\psi +\tau u_{2}(t))\frac{T_{N}D}{N^{2}}(\kappa_{3}-\kappa_{2})+\gamma (\kappa_{4}-\kappa_{5})+\omega_{2}(\kappa_{4}-\kappa_{2})(\mu +\sigma_{3})\kappa_{4}-C_{3}$$

$$\frac{d\kappa_{5}}{dt}=-\frac{\partial H_{C}}{\partial R}=\omega (\kappa_{5}-\kappa_{2})+(\psi +\tau u_{2}(t))\frac{T_{N}D}{N^{2}}(\kappa_{3}-\kappa_{2})+\kappa_{5}\mu$$ (16)

Let $\left(\tilde{S},\tilde{D},\tilde{T_{N}},\tilde{T_{P}},\tilde{R}\right)$ be optimum solution of $\left(S,D,T_{N},T_{T},R\right)$ respectively.

Let $\tilde{\kappa_{1}},\tilde{\kappa_{2}},\tilde{\kappa_{3}},\tilde{\kappa_{4}},\tilde{\kappa_{5}},$ be a solution of system (16). By using [54,55], we state and prove the below theorem.

**Theorem 4.1.** *There exist optimal controls $u_{1}^{*},u_{2}^{*},u_{3}^{*}\in \Gamma$, such that $J(u_{1}^{*},u_{2}^{*},u_{3}^{*})=\min J(u_{1},u_{2},u_{3})$ subject to extended system of equations (14).*

*Proof:* To validate this theorem, we employ [56], We note that the controls in this instance are not negative. To minimize the issue, the objective functional in $\left(u_{1},u_{2},u_{3}\right)$ must satisfy the required convexity. By definition, the collection of control variables, $u_{1},u_{2},u_{3}$ is convex. The integrand $C_{1}D+C_{2}T_{N}+C_{3}T_{T}+\frac{1}{2}C_{4}u_{1}^{2}+\frac{1}{2}C_{5}u_{2}^{2}+\frac{1}{2}C_{6}u_{3}^{2}$ of the functional *J* is convex on Γ, and its state variables are bounded. Since there are optimal controls for minimizing the convexity on Γ (14),(16) We utilize Pontryagin’s maximum principle to derive the necessary conditions to find the optimal solutions in the following manner since there are optimal controls for minimizing the functional subject of systems. Assuming that (*z*,*u*) represents the best solution to an optimal control problem, it follows that $\kappa =\kappa_{1},\kappa_{2},...\kappa_{i}$, a non-trivial vector function, must satisfy the following conditions:


$$\frac{dz}{dt}=\frac{\partial H_{C}(t,z,u,\kappa )}{dt},\;\;0=\frac{\partial H_{C}(t,z,u,\kappa )}{\partial u}\;\text{at}\;u^{⋆},\;\frac{d\kappa }{dt}=\frac{\partial H_{C}(t,z,u,\kappa )}{\partial z}.$$

**Theorem 4.2.** *The optimal controls $u_{1}^{*},u_{2}^{*},u_{3}^{*}$ which minimize J over the region Γ is given by:*


$$u_{1}^{*}=\min\{1,\max(0,\tilde{u_{1}})\},\;u_{2}^{*}=\min\{1,\max(0,\tilde{u_{2}})\},$$

$$u_{3}^{*}=\min\{1,\max(0,\tilde{u_{3}})\}$$

*where*


$$\tilde{u_{1}}=\frac{\beta (\eta \alpha_{1}+\alpha_{2})S(\kappa_{2}-\kappa_{1})}{C_{4}},\;\tilde{u_{2}}=\frac{\tau T_{N}D(\kappa_{2}-\kappa_{3})}{NC_{5}},$$

$$\;\tilde{u_{3}}=\frac{\rho D(\kappa_{2}-\kappa_{4})}{C_{6}}.$$

*Proof:* We use results in [55] and previous Theorem 4.1 to establish this theorem. Using the optimally condition: $\frac{\partial H_{C}}{\partial u_{1}}=0,\;\frac{\partial H_{C}}{\partial u_{2}}=0,\frac{\partial H_{C}}{\partial u_{3}}=0$, we get,


$$\frac{\partial H_{C}}{\partial u_{1}}=u_{1}C_{4}+\beta (\eta \alpha_{1}+\alpha_{2})S(\kappa_{1}-\kappa_{2})=0,$$

$$⟹u_{1}=\frac{\beta (\eta \alpha_{1}+\alpha_{2})S(\kappa_{2}-\kappa_{1})}{C_{4}}.$$

$$\frac{\partial H_{C}}{\partial u_{2}}=u_{2}C_{5}+\tau (\kappa_{2}-\kappa_{3})\frac{T_{N}D}{N}=0,$$

$$⟹u_{2}=\frac{\tau T_{N}D(\kappa_{2}-\kappa_{3})}{NC_{5}}.$$

$$\frac{\partial H_{C}}{\partial u_{3}}=u_{3}C_{6}+\rho D(\kappa_{2}-\kappa_{4})=0,$$

$$⟹u_{3}=\frac{\rho D(\kappa_{2}-\kappa_{4})}{C_{6}}.$$

Here 0 is the lower bound and 1 is the upper bound for the controls $u_{i}^{\prime }s$. This indicates that $u_{1}=u_{2}=u_{3}=0$, if $\tilde{u_{1}}\;<\;0,\tilde{u_{2}}<0,\tilde{u_{3}}<0$ and $u_{1}=u_{2}=u_{3}=1$ if $\tilde{u_{1}}>1;\;\tilde{u_{2}}>1;\;\tilde{u_{3}}>1;$

Hence for these optimal controls $u_{1}^{⋆},\;u_{2}^{⋆},\;u_{3}^{⋆}$ we have found the Optimum values of *J*. □

### 4.1 Cost-effectiveness analysis and numerical simulations of intervention strategies

This section presents a detailed numerical optimal control analysis performed over a 25-year period using MATLAB. The simulations employ the parameter set given in Table 1, with τ=0.35 and ρ=0.56.

The weight constants in the objective functional were chosen as follows: $C_{1}=1,C_{2}=1,C_{3}=1,C_{4}=850,C_{5}=1000,$ and *C*_6_ = 500. The values of *C*_1_, *C*_2_, and *C*_3_ were set equally to assign equal importance to minimizing the sizes of all relevant population compartments. The ordering $C_{6}<C_{4}<C_{5}$ reflects the relative cost of implementing the control measures, with pharmacological treatment considered less expensive than lifestyle-based interventions such as yoga and exercise, while also accounting for their relative significance in the model. The equations are solved iteratively using a combination of forward and backward difference approximation methods [54]. Initially, the forward difference approximation is used to solve the state equations, and then the backward difference approximation is applied to solve the adjoint equations.

A **cost-effectiveness analysis (CEA)** is conducted to assess the economic feasibility of the proposed control strategies. The analysis is performed under three distinct scenarios (Scenario I through Scenario III), each representing different assumptions or conditions for intervention:

- **Scenario I:** (Only one control strategy is applied)
  ⋄ Strategy 1: ($u_{1}\neq 0$, $u_{2}=u_{3}=0$) Implementing measures such as creating awareness among individuals to reduce the risk of developing diabetes through lifestyle changes and education.
  ⋄ Strategy 2: ($u_{2}\neq 0$, $u_{1}=u_{3}=0$) Promoting non-pharmacological treatments, including regular physical exercise, dietary modifications, and weight management, to improve glycemic control.
  ⋄ Strategy 3: ($u_{3}\neq 0$, $u_{1}=u_{2}=0$) Enhancing pharmacological treatment by increasing medication adherence, access to drugs, and timely medical interventions.
- **Scenario II:** (Two control strategies are applied)
  ⋄ Strategy 4: ($u_{1}\neq 0$, $u_{2}\neq 0$, *u*_3_ = 0) This approach focuses on raising awareness to lower diabetes risk, alongside encouraging lifestyle changes such as diet and exercise to manage the condition naturally.
  ⋄ Strategy 5: ($u_{1}\neq 0$, $u_{3}\neq 0$, *u*_2_ = 0) Combining educational efforts with increased access to and adherence to pharmacological treatments aims to improve patient outcomes through both prevention and medication.
  ⋄ Strategy 6: ($u_{2}\neq 0$, $u_{3}\neq 0$, *u*_1_ = 0) This strategy prioritizes medical treatment and lifestyle modifications together, targeting comprehensive diabetes management without specific awareness campaigns.
- **Scenario III:** (All three control strategies are applied)
  ⋄ Strategy 7: This comprehensive approach simultaneously implements awareness programs to reduce diabetes risk ($u_{1}\neq 0$), promotes non-pharmacological treatments such as lifestyle modifications ($u_{2}\neq 0$), and enhances pharmacological treatment access and adherence ($u_{3}\neq 0$) to achieve maximum control over the disease.

These scenarios enable a detailed comparison of different intervention strategies under various conditions, providing a comprehensive analysis of the cost-effectiveness and overall impact of the proposed diabetes control measures.

### 4.2 Cost-effectiveness analysis

To determine the most cost-effective control intervention strategy for each of the scenarios (I-III) analyzed in the previous section, a cost-effectiveness analysis (CEA) is performed. Three approaches are employed for this analysis: the Infection Averted Ratio (IAR) [57], the Average Cost-Effectiveness Ratio (ACER), and the Incremental Cost-Effectiveness Ratio (ICER) [57–59]. The definitions of these approaches are as follows:

**Definition 4.3.** ( **Infection Averted Ratio (IAR)**) The Infection Averted Ratio (IAR) is defined as:


$$IAR=\frac{\text{Number of infections averted}}{\text{Number of individuals recovered from the infection}}.$$

In the present scenario


$$IAR=\frac{\mathrm{Number}\mathrm{of}\mathrm{diabetes}\mathrm{cases}\mathrm{averted}}{\mathrm{Number}\mathrm{of}\mathrm{individuals}\mathrm{controlled}\mathrm{diabetes}},$$

where the number of diabetes cases averted is the difference between the total number of diabetes cases in the absence of any control measures and the total number of diabetes cases under the implemented control strategy. The strategy with the highest IAR value is considered the most cost-effective [57,59,60].

**Definition 4.4.** ( **Average Cost-Effectiveness Ratio (ACER)**) The Average Cost-Effectiveness Ratio (ACER) is given by:


$$ACER=\frac{\mathrm{Total}\mathrm{cost}\mathrm{associated}\mathrm{with}\mathrm{implementing}a\mathrm{specific}\mathrm{intervention}\mathrm{strategy}}{\mathrm{Total}\mathrm{number}\mathrm{of}\mathrm{cases}\mathrm{prevented}\mathrm{by}\mathrm{the}\mathrm{intervention}\mathrm{strategy}},$$

where the total cost incurred in executing a particular intervention strategy is computed as:


$$C(u)=\frac{1}{2}\int_{0}^{T}\sum_{i=1}^{3}C_{i}u_{i}^{2}\;dt.$$

**Definition 4.5. (Incremental Cost-Effectiveness Ratio (ICER))**

Incremental Cost-Effectiveness Ratio (ICER) measures the change in costs and health benefits between two different intervention strategies competing for the same limited resources.

Considering strategies *p* and *q* as two competing control intervention strategies, ICER is defined as:


$$ICER=\frac{\mathrm{Change in total costs between strategies}p\mathrm{and}q}{\mathrm{Change in control benefits between strategies}p\mathrm{and}q}$$

This process is repeated progressively for all strategies. A strategy with higher costs but fewer benefits is considered dominant and is eliminated from further analysis. The final ICER values help identify the most cost-effective strategy among the three, providing a basis for decision-making in resource allocation.

#### 4.2.1 Scenario-I.

**Fig 16:**
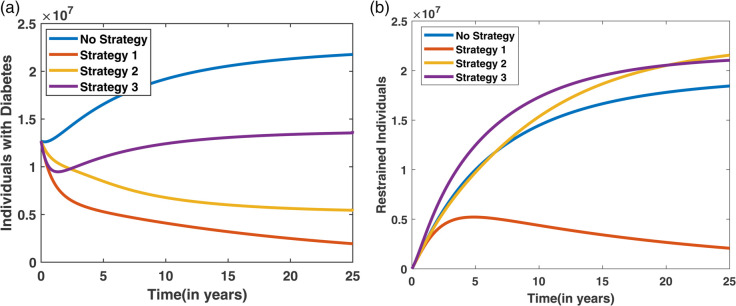
Effect of individual control strategies on diabetes dynamics: (a) shows the variation in the diabetic population under different strategies, with Strategy 1 resulting in the lowest diabetes burden; (b) illustrates the restrained population, where Strategy 3 leads to the highest number individuals in the restrained (controlled) state.

**Fig 17:**
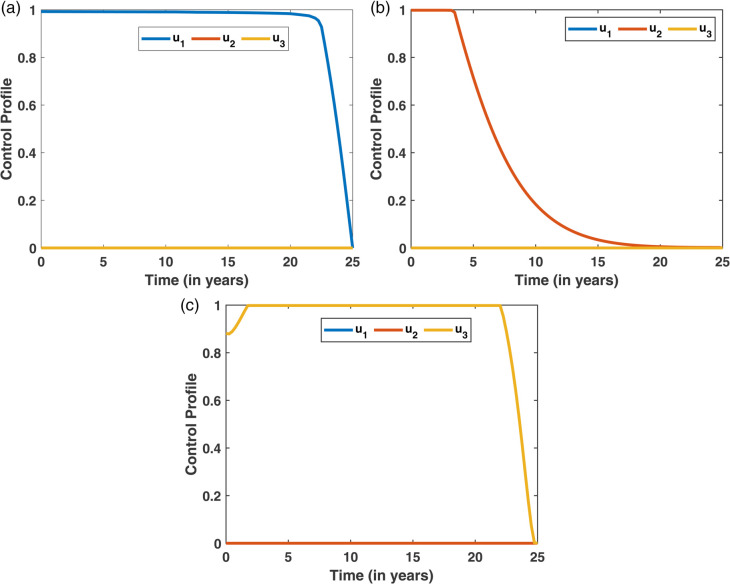
Control profiles corresponding to the three strategies. The control *u*_2_ (non-pharmacological treatment) is maintained over a longer period, highlighting its sustained importance in effective diabetes management.

In this scenario, each control measure is applied individually to evaluate its independent impact on diabetes management. This helps identify the effectiveness and significance of each strategy before exploring combined interventions. Fig 16 illustrates the dynamics of the diabetes and restrained compartments. In Fig 16(a), which represents individuals in the diabetes compartment, the number of diabetes cases varies across different strategies, with Strategy 1 resulting in the lowest diabetes count. Similarly, the Fig 16(b) shows the restraint compartment, where strategy 3 plays an important role in increasing the number of restrained individuals. Fig 17 presents the control profiles for the three strategies, indicating that the *u*_2_ control (non-pharmacological treatment) needs to be maintained for a longer duration to be effective.

**Fig 18:**
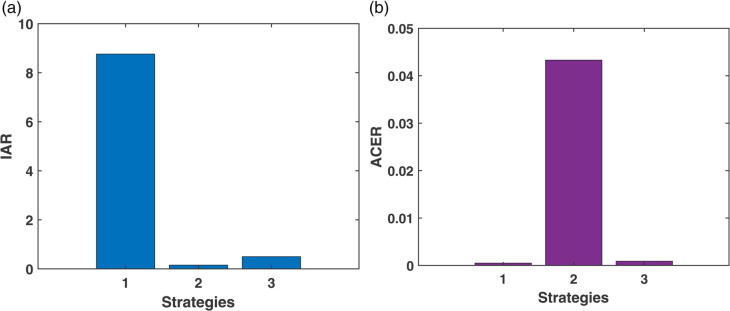
IAR and ACER Analysis for Scenario-I.

The Incremental Cost-Effectiveness Ratio (ICER) analysis is presented using six columns in Table 5, which include the strategy, diabetes cases averted, cost, IAR (Incremental Averted Ratio), ACER (Average Cost-Effectiveness Ratio), and ICER values. The strategies are first arranged in ascending order based on the number of diabetes cases averted, and their corresponding IAR, ACER, and ICER values are computed. From Table 5, it is observed that Strategy 1 has a higher IAR and a lower ACER, suggesting that Strategy 1 is more effective compared to the other individual strategies. This effectiveness is also visualized in Fig 18. Furthermore, Strategy 3 exhibits the highest ICER value, indicating it is less cost-effective and, therefore, excluded from further analysis. A refined comparison is then presented in Table 6, where Strategy 1 shows a lower ICER compared to Strategy 2. Thus, based on the IAR, ACER, and ICER metrics, Strategy 1 is identified as the most effective option to control diabetes under Scenario I, which is also shown in Fig 16.

**Table 5:** ICER(Incremental cost-effectiveness ratio) for scenario I.

Strategy	Diabetes cases averted	Cost	IAR	ACER	ICER
2	$2.80\times 10^{5}$	12120.62	0.0154	0.0433	0.0433
3	$6.311\times 10^{6}$	6164.56	0.2996	0.0009	-0.0009
1	$1.960\times 10^{7}$	10544.22	8.7622	0.0005	0.0003

**Table 6:** ICER (Incremental cost-effectiveness ratio) for scenario I.

Strategy	Diabetes cases averted	Cost	IAR	ACER	ICER
3	$6.311\times 10^{6}$	6164.56	0.2996	0.0009	0.0009
1	$1.960\times 10^{7}$	10544.22	8.7622	0.0005	**0.0003**

#### 4.2.2 Scenario-II.

**Fig 19:**
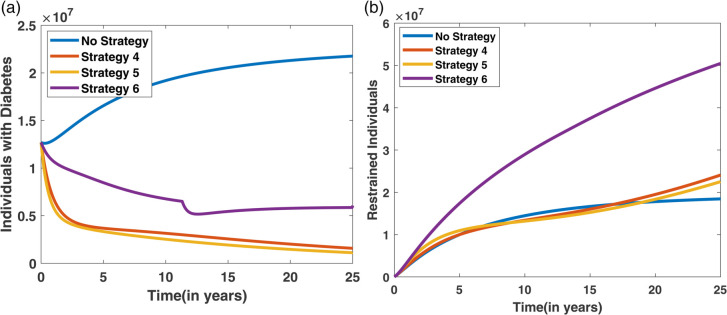
Effect of two combined control strategies on diabetes dynamics: (a) depicts the diabetic population, with Strategy 4 yielding the lowest number of cases; (b) shows the restrained population, where Strategy 6 achieves the highest restrain level.

**Fig 20:**
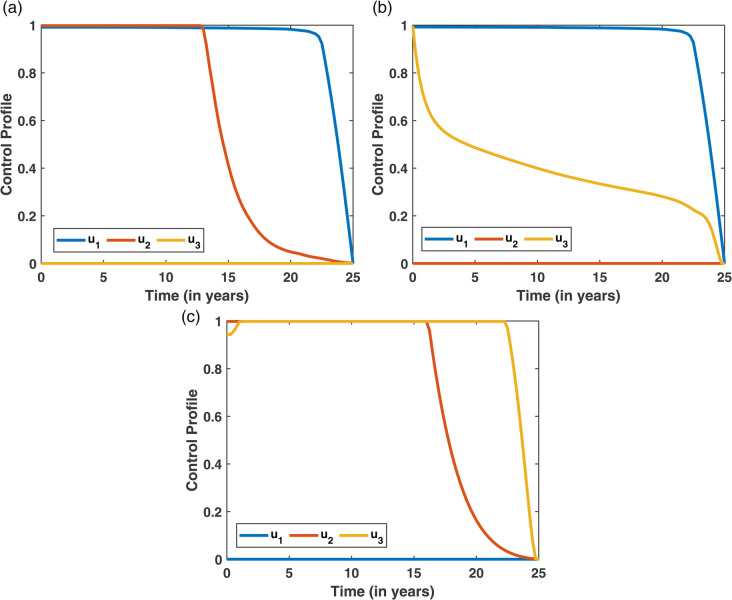
Control profiles corresponding to the three strategies.

In Scenario II, two control strategies are applied together to assess their combined impact on diabetes dynamics. This helps determine whether paired interventions are more effective than using each control individually. Fig 19 displays the progression of both the diabetes and restrained compartments under the application of two combined control strategies. Fig 19(a) highlights the trends in the diabetic population, revealing that Strategy 5 leads to the most significant reduction in diabetes cases. In contrast, Fig 19(b) shows that Strategy 6 contributes most effectively to boosting the number of restrained individuals. Meanwhile, the control effort trends are depicted in Fig 20, where it is evident that Strategy 5 requires sustained implementation over a longer period to achieve meaningful impact.

**Fig 21:**
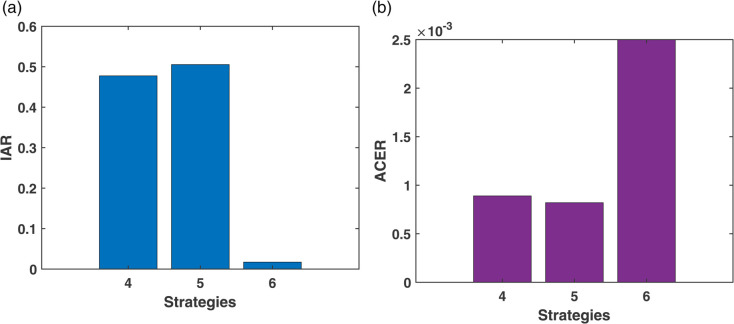
IAR and ACER Analysis for Scenario-II.

The cost-effectiveness assessment for Scenario II is presented in Table 7, which outlines key metrics including the number of diabetes cases averted, total cost, Incremental Averted Ratio (IAR), Average Cost-Effectiveness Ratio (ACER), and Incremental Cost-Effectiveness Ratio (ICER) for each strategy. The strategies are arranged in order of increasing diabetes cases averted, and corresponding values for IAR, ACER, and ICER are calculated. With a lower ACER and a higher IAR, Strategy 5 exhibits a favorable balance and is therefore more cost-effective than the other strategies, as shown in Table 7. This trend is visualized in Fig 21. In contrast, Strategy 6 has the highest ICER, making it the least efficient and subsequently excluded from further evaluation. A focused comparison between the remaining strategies in Table 8 reveals that Strategy 5 continues to outperform, with a lower ICER than Strategy 4. Therefore, Strategy 5 emerges as the most cost-effective intervention in Scenario II for managing diabetes, which is also shown in Fig 19.

**Table 7:** ICER(Incremental cost-effectiveness ratio) for scenario II.

Strategy	Diabetes cases averted	Cost	IAR	ACER	ICER
6	$6.365\times 10^{6}$	16082.68	0.3035	0.0025	0.0025
4	$1.963\times 10^{6}$	17559.755	8.4375	0.00089	0.0001
5	$2.032\times 10^{6}$	16708.78	8.9295	0.00082	–0.0012

**Table 8:** ICER(Incremental cost-effectiveness ratio) for scenario II.

Strategy	Diabetes cases averted	Cost	IAR	ACER	ICER
4	$1.963\times 10^{6}$	17559.755	8.4375	0.00089	0.00089
5	$2.032\times 10^{6}$	16708.78	8.9295	0.00082	**–0.0012**

#### 4.2.3 Scenario-III.

**Fig 22:**
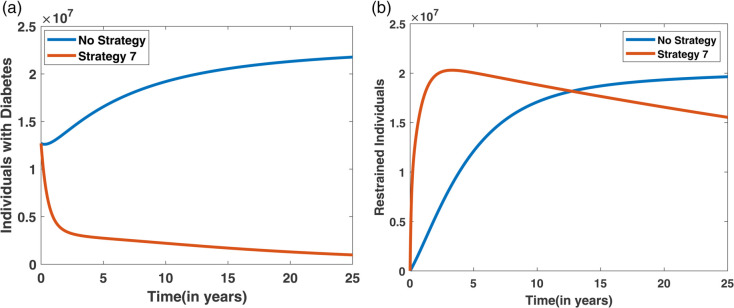
Effect of Strategy 7 (all controls applied) on diabetes and restrain dynamics compared to the no-control scenario.

**Fig 23:**
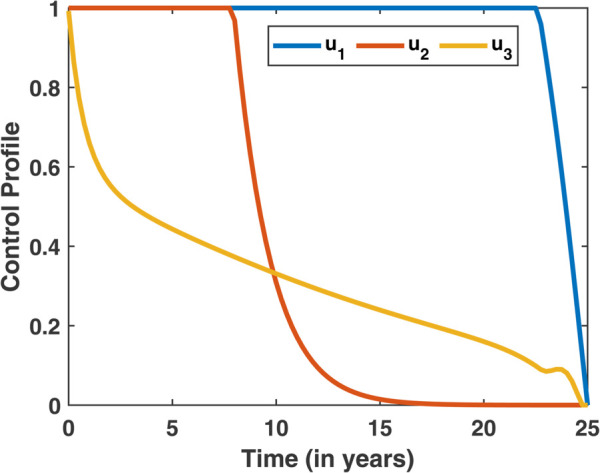
Contour profiles of control measures for Strategy 7 over time.

The simulation results for the optimal control system under Scenario III, where Strategy 7 (all three control measures applied simultaneously) is implemented, are illustrated in Fig 22. A comparison between the no-control case and Strategy 7 demonstrates a notable reduction in the number of diabetes individuals and a corresponding increase in the number of individuals entering the restrained state when the strategy is applied. Additionally, the cost-effectiveness of Strategy 7 is evaluated using the Incremental Averted Ratio (IAR) and Average Cost-Effectiveness Ratio (ACER), with the outcomes summarized in Table 9. Fig 23 presents the contour profile of Strategy 7, illustrating the intensity and duration of the control measures over time.

**Table 9:** ICER(Incremental cost-effectiveness ratio) for scenario III.

Strategy	Diabetes cases averted	Cost	IAR	ACER
7	$2.0526\times 10^{7}$	22526.57	9.5028	0.0011

### 4.3 Identifying the strategy with the best cost-effectiveness

After evaluating the performance of all control strategies within their respective scenarios, we proceed to a comprehensive comparison of the most effective strategies identified: Strategy 1 from Scenario I, Strategy 5 from Scenario II, and Strategy 7 from Scenario III. These selected strategies are further analyzed to determine the most cost-effective approach using the Incremental Cost-Effectiveness Ratio (ICER) methodology.

For each strategy pair, ICER values are calculated as previously described. Any strategy that is dominated meaning it incurs higher costs with comparatively fewer health benefits is excluded from further analysis. According to Table 10, Strategy 7 is found to be less cost-effective and is subsequently removed from consideration. The refined comparison in Table 11 indicates that Strategy 1 offers the best balance of effectiveness and cost efficiency.

**Table 10:** Incremental Cost-Effectiveness Ratio (ICER) for optimal strategies across scenarios.

Scenario	Strategy	Diabetes cases averted	Cost	IAR	ACER	ICER
I	1	$1.96\times 10^{7}$	10544.22	8.7622	0.0005	0.0005
II	5	$2.0326\times 10^{7}$	16708.78	8.9295	0.00082	0.0085
III	7	$2.0526\times 10^{7}$	22526.57	9.5028	0.0011	0.0291

**Table 11:** Incremental Cost-Effectiveness Ratio (ICER) for optimal strategies across scenarios.

Scenario	Strategy	Diabetes cases averted	Cost	IAR	ACER	ICER
I	1	$1.96\times 10^{7}$	10544.22	8.7622	0.0005	**0.0005**
II	5	$2.0326\times 10^{7}$	16708.78	8.9295	0.00082	0.0085

This analysis underscores the value of the control *u*_1_ which emphasizes awareness creation and early interventions. It proves to be the most impactful and economically viable strategy for managing diabetes.

## 5 Results and discussion

Diabetes is one of the major chronic diseases, causing severe illness and long-term health complications. In this study, we incorporated obesity as one parameter, and genetic and other complications as another, such that their sum equals one. Two types of treatment were considered: non-pharmacological (e.g., lifestyle modification, yoga, exercise) and pharmacological (e.g., proper medication, insulin administration).

An important behavioral assumption in the model is that individuals following non-pharmacological treatment who controlled or successfully manage diabetes inspire their friends and neighbors to adopt similar strategies. This peer influence is modeled through a mass action term $\left(\frac{\psi DT_{N}}{N}\right)$ representing the transition from the diabetic compartment (*D*) to the non-pharmacological treatment compartment (*T*_*N*_). The proposed compartmental model for diabetes exhibits three equilibrium points: the diabetes-free equilibrium (*E*_0_), the diabetic equilibrium without non-pharmacological treatment (*E*_1_), and the endemic equilibrium (*E*_2_). We established the local and global stability of these equilibria both analytically and graphically.

The model was fitted to real-world diabetes data from the United States spanning 2000 to 2022, and the model parameters were estimated accordingly. Partial Rank Correlation Coefficient (PRCC) sensitivity analysis was conducted for the compartments *D*, *T*_*N*_, *T*_*P*_, and *R* to identify the most influential parameters. The parameters β,ψ,θ,Λ,δ,γ, and *ω* were found to be particularly sensitive, indicating their critical roles in disease control and the promotion of diabetes restrain.

Further investigation using contour plots revealed that increasing the parameters *ψ* (rate of initiating non-pharmacological treatment) and *δ* (rate of initiating pharmacological treatment) significantly reduces the diabetic population. Conversely, parameters such as $\beta ,\alpha_{1},$ and $\alpha_{2}$ contribute to the increase in diabetic individuals, with $\alpha_{1}$ having a more pronounced effect than $\alpha_{2}$. Parameters ψ,δ,θ, and *γ* were found to promote recovery and restraint from diabetes.

In addition to these, the relapse and treatment failure parameters also played a critical role in shaping long-term disease outcomes. The parameter *ω* (relapse after restrain) was found to be the most influential among all failure pathways, producing a substantial increase in the diabetic population even when treatment options are available. Treatment failure from non-pharmacological and pharmacological interventions, represented by $\omega_{1}$ and $\omega_{2}$ respectively, also increased the diabetic burden, but their impacts were comparatively weaker than relapse. These results emphasise that preventing relapse and sustaining treatment adherence are more crucial than simply increasing treatment intensity. Continuous follow-up, lifestyle maintenance, and behavioural reinforcement are therefore essential to achieve durable control.

To ensure reliable numerical solutions, we compared the Forward Euler, RK4, and NSFD methods. The NSFD scheme demonstrated superior stability and accuracy, particularly for larger step sizes, where Euler and RK4 methods tended to diverge. It preserved convergence, boundedness, and the system’s qualitative behavior, confirming its robustness through error analysis. Overall, the NSFD method proved to be a reliable tool for simulating chronic disease dynamics.

The study was extended to include optimal control and cost-effectiveness analysis by introducing three controls: *u*_1_ (awareness campaigns), *u*_2_ (promotion of non-pharmacological treatment), and *u*_3_ (promotion of pharmacological treatment). Three scenarios were considered:

- **Scenario I**: Only one control applied. Strategy 1 (*u*_1_ only) was found to be the most effective.
- **Scenario II**: Two controls applied. Strategy 5 (combining *u*_1_ and *u*_3_) was superior among the three strategies.
- **Scenario III**: All three controls applied.

Ultimately, cost-effectiveness analysis indicated that **Scenario I**, with only awareness control (*u*_1_), is the most cost-effective strategy for managing diabetes.

## 6 Conclusion

Diabetes remains a major chronic disease with severe global health and economic impacts. This study developed and analysed a compartmental model incorporating both pharmacological and non-pharmacological interventions, with peer influence modelled through a mass–action mechanism. Stability of the three equilibrium points was established analytically and numerically.

Model fitting to U.S. prevalence data (2000–2022) and sensitivity analysis highlighted the importance of treatment uptake and lifestyle adoption in reducing the diabetic population. Numerical results confirmed the reliability of the NSFD method compared to Euler and RK4. Optimal control analysis further showed that awareness campaigns, particularly when combined with lifestyle promotion, represent the most cost-effective intervention.

Overall, the findings underscore the need for preventive strategies and timely medical treatment to curb diabetes prevalence. Moreover, our analysis indicates that relapse (*ω*) and treatment failure rates ($\omega_{1}$, $\omega_{2}$) substantially sustain the long-term diabetic burden, emphasizing the necessity of continued follow-up, adherence support, and prevention of treatment discontinuation even after initial controlled stage. Future extensions may consider stochastic effects, fractional-order models, or co-morbidity for a more comprehensive framework.

## Supporting information

S1 File Appendix. This file contains the mathematical proofs of Theorems 1.1, 1.2, and 1.3. (PDF)
