# Supplementary material for: Mathematical modeling and cost-effective intervention strategies for diabetes management: A data-driven and numerical analysis approach
Source: PLoS One. 2026 Jan 16;21(1):e0339463. doi: 10.1371/journal.pone.0339463 (PMC12810924; doi:10.1371/journal.pone.0339463)
Supplement: S1 File — (PDF) [file pone.0339463.s001.pdf]

1 Supporting information

S1. Proof of Theorem 1.1

Proof. To analyze stability, we compute the Jacobian matrix J_{E_0} of system (1) at the diabetes-free equilibrium E_0:

$$J_{E_0} = \begin{pmatrix} -(A_1+\mu) & 0 & 0 & 0 & 0 \\ A_1 & -A_2 & \omega_1 & \omega_2 & \omega \\ 0 & 0 & -A_3 & 0 & 0 \\ 0 & \delta & \phi & -A_4 & 0 \\ 0 & 0 & \theta & \gamma & -A_5 \end{pmatrix}$$

The characteristic equation $|J_{E_0} - MI| = 0$ is given by:

$$(M + A_1 + \mu)(M + A_3)\left(M^3 + C_1 M^2 + C_2 M + C_3\right) = 0,$$

where

$$\begin{aligned} C_1 &= A_2 + A_4 + A_5, \\ C_2 &= A_5A_2 + A_4A_5 + A_2A_4 + \delta\omega_2, \\ C_3 &= A_4A_2A_5 + \delta\omega_2A_5 - \delta\gamma\omega \\ &= \delta\gamma\mu + \delta(\omega_2 + \mu + \sigma_3)A_5 + (\mu + \sigma_1)A_4A_5. \end{aligned}$$

Hence, the diabetes-free equilibrium E_0 is locally asymptotically stable if the Routh–Hurwitz conditions for the cubic polynomial are satisfied:

$$C_1 > 0, \quad C_3 > 0, \quad C_1C_2 - C_3 > 0.$$

□

S2.Proof of Theorem 1.2

Proof. To analyze stability, we compute the Jacobian matrix J_{E_1} of system (1) at the diabetic equilibrium without non-pharmacological treatment E_1:

$$J_{E_1} = \begin{pmatrix} -(A_1+\mu) & 0 & 0 & 0 & 0 \\ A_1 & -A_2 & \omega_1 - \dfrac{\psi D}{N_1} & \omega_2 & \omega \\ 0 & 0 & \dfrac{\psi D}{N_1} - A_3 & 0 & 0 \\ 0 & \delta & \phi & -A_4 & 0 \\ 0 & 0 & \theta & \gamma & -A_5 \end{pmatrix}$$

The characteristic equation $|J_{E_1} - XI| = 0$ is given by:

$$(X + \beta(\eta\alpha_1 + \alpha_2) + \mu)\left(X + A_3 - \frac{\psi D}{N_1})\right)\left(X^3 + D_1X^2 + D_2X + D_3\right) = 0,$$

where

$$\begin{aligned} N_1 &= S + D + T_P + R, \\ D_1 &= A_2 + A_4 + A_5, \\ D_2 &= A_5A_2 + A_4A_5 + A_2A_4 + \delta\omega_2, \\ D_3 &= A_4A_2A_5 + \delta\omega_2A_5 - \delta\gamma\omega \\ &= \delta\gamma\mu + \delta(\omega_2 + \mu + \sigma_3)A_5 + (\mu + \sigma_1)A_4A_5. \end{aligned}$$

The two linear roots are clearly negative if

$$\frac{\psi D}{N_1} < A_3.$$

Thus, the diabetic equilibrium without non-pharmacological treatment E_1 is locally asymptotically stable if the Routh–Hurwitz conditions for the cubic polynomial are satisfied:

$$D_1 > 0, \quad D_3 > 0, \quad D_1 D_2 - D_3 > 0.$$

□

S3.Proof of Theorem 1.3

Proof. To analyze stability, we compute the Jacobian matrix J_{E_2} of system (1) at the endemic equilibrium E_2:

$$J_{E_2} = \begin{pmatrix} -(A_1+\mu) & 0 & 0 & 0 & 0 \\ A_1 & -(A_2 + \frac{\psi T_N}{N^2}(N-D)) & \omega_1 - \frac{\psi D}{N^2}(N-T_N) & \omega_2 & \omega \\ 0 & \frac{\psi T_N}{N^2}(N-D) & \frac{\psi D}{N^2}(N-T_N) - A_3 & 0 & 0 \\ 0 & \delta & \phi & -A_4 & 0 \\ 0 & 0 & \theta & \gamma & -A_5 \end{pmatrix}$$

The characteristic equation $|J_{E_2} - YI| = 0$ is given by:

$$(Y + \beta(\eta\alpha_1 + \alpha_2) + \mu)\left(Y^4 + G_1 Y^3 + G_2 Y^2 + G_3 Y + G_4\right) = 0,$$

where

$$\begin{aligned} g_1 &= \frac{\psi T_N}{N^2}(N-D), \quad g_2 = \frac{\psi D}{N^2}(N-T_N), \\ G_1 &= A_4 + A_5 + A_2 + A_3 + g_1 - g_2, \\ G_2 &= (A_3 - g_2)(A_4 + A_5 + A_2 + g_1) + A_4A_5 + (A_2 + g_1)(A_4 + A_5) + g_1(g_2 - \omega_1), \\ G_3 &= (A_3 - g_2)(A_4A_5 + (A_4 + A_5)(A_2 + g_1)) + A_4A_5(A_2 + g_1) + g_1(g_2 - \omega_1)(A_4 + A_5) \\ &\quad - g_1(\phi\omega_2 + \theta\omega), \\ G_4 &= (A_3 - g_2)(A_2 + g_1)A_4A_5 + g_1(g_2 - \omega_1)A_4A_5 - g_1A_4\theta\omega. \end{aligned}$$

The root $Y = -(\beta(\eta\alpha_1 + \alpha_2) + \mu)$ is clearly negative. Thus, the local asymptotic stability of E_2 depends on the quartic polynomial.

By the Routh–Hurwitz criteria for a fourth-degree polynomial, the equilibrium E_2 is locally asymptotically stable if the following conditions hold:

$$\begin{aligned} &G_1 > 0, \\ &G_1G_2 - G_3 > 0, \\ &(G_1G_2 - G_3)G_3 - G_1^2 G_4 > 0, \\ &G_4 > 0. \end{aligned}$$

□
